# Blockchain Technology Secures Robot Swarms: A Comparison of Consensus Protocols and Their Resilience to Byzantine Robots

**DOI:** 10.3389/frobt.2020.00054

**Published:** 2020-05-12

**Authors:** Volker Strobel, Eduardo Castelló Ferrer, Marco Dorigo

**Affiliations:** ^1^IRIDIA, Université Libre de Bruxelles, Brussels, Belgium; ^2^MIT Media Lab, Cambridge, MA, United States

**Keywords:** swarm robotics, blockchain technology, Byzantine fault-tolerance, resilient robotics, verifiable robotics

## Abstract

Consensus achievement is a crucial capability for robot swarms, for example, for path selection, spatial aggregation, or collective sensing. However, the presence of malfunctioning and malicious robots (Byzantine robots) can make it impossible to achieve consensus using classical consensus protocols. In this work, we show how a swarm of robots can achieve consensus even in the presence of Byzantine robots by exploiting blockchain technology. Bitcoin and later blockchain frameworks, such as Ethereum, have revolutionized financial transactions. These frameworks are based on decentralized databases (blockchains) that can achieve secure consensus in peer-to-peer networks. We illustrate our approach in a collective sensing scenario where robots in a swarm are controlled via blockchain-based smart contracts (decentralized protocols executed via blockchain technology) that serve as “meta-controllers” and we compare it to state-of-the-art consensus protocols using a robot swarm simulator. Additionally, we show that our blockchain-based approach can prevent attacks where robots forge a large number of identities (Sybil attacks). The developed robot-blockchain interface is released as open-source software in order to facilitate future research in blockchain-controlled robot swarms. Besides increasing security, we expect the presented approach to be important for data analysis, digital forensics, and robot-to-robot financial transactions in robot swarms.

## 1. Introduction

Disasters, such as the collapse of a nuclear plant (e.g., Fukushima) or the release of petroleum into the environment (e.g., the Deepwater Horizon oil spill), present huge challenges and require quick and efficient responses. For example, it might be crucial to determine the average presence of radiation in a contaminated area (Brown et al., 2016). For security and efficiency reasons, on-site intervention might be better delegated to autonomous robots; and, to make the response more effective and mitigate potential adverse effects, the robots might have to perceive and act in different places at the same time. The coordination of such distributed activities by a central unit of control is not ideal as it makes the system less reliable (single point of failure) and possibly less efficient (communication overheads, delay in the collection of data, and in the release of control commands). Robot swarms, that communicate and collaborate in a peer-to-peer manner, are excellent candidates for these situations.

One important capability that robot swarms need to have to cooperate effectively is to be able to make collective decisions. Accordingly, collective decision-making is a well-studied subject in the field of swarm robotics (Schmickl et al., 2009; Montes de Oca et al., 2011; Reina et al., 2015; Valentini et al., 2016b, 2017). In general, to make a collective decision, robots in a swarm need to share their information and to aggregate this information using a distributed consensus protocol. The prevailing consensus protocol for averaging the values held by the individual entities in the swarm is the linear consensus protocol (lcp) (Olfati-Saber and Murray, 2004). However, this consensus protocol and most other protocols used in swarm robotics make the unrealistic assumption that all the robots in the swarm work as expected.

Unfortunately, real-world operation will almost certainly result in robots in the swarm that either fail (e.g., due to dust blocking their sensors) or that are malicious (e.g., due to a hacker who gains control). These failures can damage people, nature, animals, and other robots, making the reliable detection of failures a crucial task (Tarapore et al., 2019). We use the term Byzantine robot—based on Byzantine fault-tolerance and the *Byzantine Generals Problem* (Lamport et al., 1982)—as an umbrella term to describe robots that show unintended or inconsistent behavior, independent of the underlying cause. A Byzantine robot can appear well-functioning to some part of the swarm but faulty to others and might arbitrarily change its behavior. An extension of the lcp capable of managing these Byzantine robots is the weighted-mean-subsequence-reduced (w-msr) algorithm (LeBlanc et al., 2013). While w-msr's outlier detection limits the influence of Byzantine robots as long as their number is low, it breaks down as soon as their number is high or an attacking robot forges pseudo-identities (Sybil attack).

To pave the way for real-world deployments, *secure* robot swarms must continue to operate effectively in the presence of Byzantine robots, potentially performing Sybil attacks. Peer-to-peer networks are particularly prone to Sybil attacks: without a trusted system, it is easy for a malicious agent to create an unlimited number of new identities and gain a disproportionate amount of power in the swarm (Douceur, 2002). We contend that *blockchain technology* can be used to create such secure robot swarms due to its decentralized nature, resilience, and versatility. Blockchain technology was originally developed for Bitcoin (Nakamoto, 2008), the first widely successful digital peer-to-peer currency. In the context of Bitcoin, the blockchain presents a tamper-proof financial ledger in a network of mutually untrusting agents without relying on a central authority. The Ethereum framework (Buterin, 2014) further demonstrated that the blockchain cannot only be used for financial transactions but can store snippets of programming code and come to an agreement regarding their outcome. These snippets of programming code are called blockchain-based smart contracts (or *smart contracts* for short). Every node (robot in this article) in the network runs a virtual machine and executes these snippets. We show how smart contracts can provide the infrastructure for implementing secure “meta-controllers” in robot swarms.

*Blockchain-based meta-controller*: We define a blockchain-based meta-controller to be a controller that coordinates the swarm at a higher level than the local controllers of the individual robots. To this end, crucial information from the individual robots is securely stored, aggregated, and processed via a smart contract residing on the blockchain. This ensures that information or control commands are based on a consensus in the swarm.

We release our developed framework as open-source software. It facilitates blockchain research in swarm robotics by providing an interface between the robot swarm simulator ARGoS (Pinciroli et al., 2012) and the blockchain framework Ethereum.

In this article, we study whether robot swarms need blockchain technology. To this end, we formulate the following research questions:

- RQ 1: Can smart contracts be used to replace existing consensus protocols in robot swarms?
- RQ 2: Can smart contracts be used to mitigate the effect of Byzantine robots in robot swarms?
- RQ 3: Can smart contracts introduce scarce resources into robot swarms and prevent Sybil attacks?

**Figure 1:**
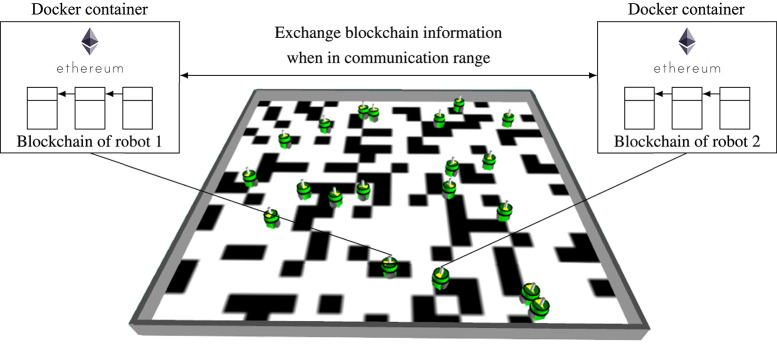
The robots' task is to determine the relative frequency of white tiles in an environment in which the floor is covered with black and white tiles. For each robot, an instance of the Ethereum blockchain software is executed in a separate Docker container and the robots maintain a custom Ethereum blockchain network. Via a blockchain-based smart contract, the sensor readings of the robots are stored and aggregated. When robots are within communication range, they exchange their blockchain information. In contrast to classical approaches, the blockchain is able to mitigate the negative impact of malfunctioning or malicious robots and allows the creation of a *tamper-proof* system, in which the messages of the robots are securely stored.

To address these research questions, we compare the two existing protocols lcp and w-msr to our blockchain-based approach in a collective decision-making scenario (Figure 1) where the robot swarm moves on a floor covered with black and white tiles and has to determine the relative frequency of the white tiles in an ARGoS environment. The scope of this study is strictly limited to swarm robotics, where global communication is not available.

The remainder of this paper is structured as follows. Section 2 summarizes the fundamentals of blockchain technology. Section 3 reviews related work in consensus achievement, security issues, and blockchain-controlled robot swarms. Section 4 lays the foundation for practical implementations by describing the ARGoS-blockchain interface. Section 5 describes the general framework for conducting the simulations in ARGoS and the technical aspects of the used consensus protocols. Section 6 presents and discusses the results of five sets of simulations—in the presence and absence of Byzantine robots. Section 7 extends the discussion to robustness, feasibility, and scalability and draws directions for future work. Section 8 presents the conclusions.

## 2. Fundamentals of Blockchain Technology for Swarm Robotics

This section summarizes the main characteristics of blockchain technology (section 2.1) and explains blockchain-based smart contracts (section 2.2).

### 2.1. General Foundation

**Figure 2:**
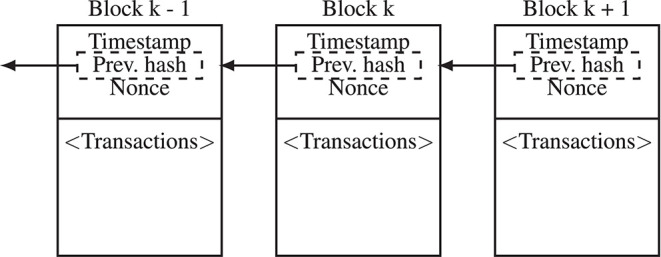
A blockchain is composed of linked blocks containing data consisting of transactions. Each block is divided into two parts: a body and a header. In the body, the transactions of the participants are stored. The header contains metadata and links each block to the hash of a previous block to create a chain of blocks. A copy of the blockchain is stored by each participant in the peer-to-peer network; the peers exchange and update their blockchain information based on a consensus protocol.

Blockchains are databases and computing platforms that are replicated and shared among the participants (robots in this work) of a peer-to-peer network (Figure 2). The pseudonymous Satoshi Nakamoto originally devised the blockchain to record digital coin transactions (transactions of cryptotokens) of the cryptocurrency Bitcoin (Nakamoto, 2008). Shortly after, there have been proposals to use the decentralized ledger for other specific, non-financial applications, such as voting, identity management, and supply chain management (Crosby et al., 2016). In 2014, Ethereum further generalized these use cases and released a framework for storing and executing programming code via blockchain technology (*blockchain-based smart contracts*) based on a Turing-complete programming language.

To interact with a blockchain and store new data, participants create transactions and distribute them among their peers. Examples of transactions are: “Send 5 ether (Ethereum's cryptocurrency) from digital address *A* to *B*” or “Execute function *X* using *Y* as input.” A transaction is digitally signed by the sender using a private key. Hence, all transactions can be unambiguously assigned to a digital address (public key) and attackers cannot create transactions under a false digital identity. In most blockchain frameworks, all data is public and can be read by every participant of the network. Still, in blockchains without an access control layer (public blockchains or permissionless blockchains), the real identities of entities (persons, organizations, robots) involved in a transaction can remain unknown since only the public keys are visible.

**Figure 3:**
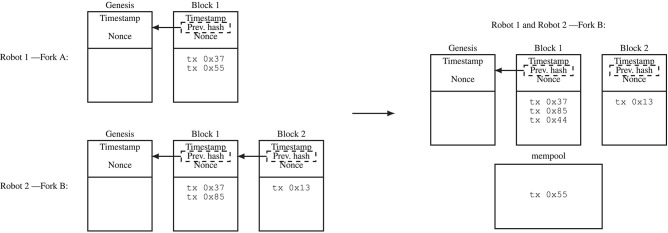
In the illustration, Robot 1 (Fork A) and Robot 2 (Fork B) have conflicting blockchain versions (forks). This situation can occur if there is a delay in communication, for example, because the two robots were in different “clusters” that could not communicate with each other and now they can communicate with each other again. Fork B is the longer blockchain (the one that contains more PoW) and is accepted as the true blockchain, while the shorter blockchain is discarded. That is, after exchanging their blockchain information, the two robots agree on Fork B. Transactions in the shorter blockchain become unconfirmed transactions again (stored in a separate memory pool) and can be included in a later block (e.g., Block 3). The memory pool contains transactions that can be included into blocks.

For a transaction to become part of the blockchain, it has to be bundled into a block and added to the end of the chain of blocks. Before being part of a block, transactions are called *unconfirmed* transactions and are disseminated across nodes of the blockchain network. Bitcoin introduced a consensus protocol which allows the participants in the network to agree on which blocks to add and in what order to add them. The consensus protocol used by Bitcoin is called Proof-of-Work (PoW) and was the first protocol to effectively reach decentralized consensus preventing at the same time double-spending (i.e., a situation where the same cryptotoken is spent twice). PoW requires the participants to solve a computational puzzle in order to add a block to the blockchain; the puzzle consists of finding a hash value below a target value using the bundled transactions and an adjustable nonce value as input to the hash function. The nonce is a number that can be arbitrarily varied in order to change the input to the hash function and, therefore, the result of the hash function. The process of solving this puzzle (i.e., modifying the nonce value given a list of transactions and calculating the resulting hash values) is called *mining*. The number of hashes a device can compute per second is stated by its hash power. Miners are motivated to perform the PoW since the first one that finds a solution to the puzzle can append the corresponding block to the blockchain and as a consequence is rewarded by immutable cryptotokens stored on the blockchain. Due to delays in the communications between the network participants, the participants can have conflicting blockchain versions (*forks*). For example, during the experiments conducted in the scope of this research, the information written in the blockchain differs among the robots that are not in communication range. However, via the PoW-based consensus protocol, conflicting blockchain versions can be resolved: whenever a robot has to choose between possible blockchains, the blockchain that required the highest PoW (i.e., the longest blockchain) gets accepted as the true blockchain, while shorter blockchains are discarded. Transactions that were in the discarded blockchains but not in the longest blockchain become unconfirmed transactions again and can be included in later blocks (Figure 3).

### 2.2. Blockchain-Based Smart Contracts

A blockchain-based smart contract (or a *smart contract* for short) is programming code that encapsulates variables and functions and is stored on the blockchain. To create a smart contract or call its functions, one needs to create a transaction and distribute it in the blockchain network. The nodes in the blockchain network keep track of the internal state (e.g., value of variables) and execute the computations of smart contracts, e.g., via the Ethereum Virtual Machine (EVM). While there are now multiple blockchain-based smart contract platforms, Ethereum remains the platform with the largest user base and the most mature technical setup.

Smart contracts were originally devised by Szabo (1997) to enforce contractual agreements between parties via computer protocols. Szabo's theoretical notion was made practically possible for the first time by the Ethereum framework: via a blockchain-based smart contract, a certain event can trigger an unstoppable financial transaction (programmable payment). However, blockchain-based smart contracts are not limited to programmable payments and the term smart contract is now used to describe any computer program that is executed on a blockchain.

For example, an Ethereum smart contract could provide the functions for selecting the winner of a talent show on TV. The audience has the possibility to vote for their favorite candidate (Alice or Bob) by sending a transaction (e.g., including 0.01 ether) to the TV's station smart contract. The smart contract on the public Ethereum blockchain keeps track of the number of votes for both candidates. Moreover, it specifies the following programmable payment: if the number of votes for one candidate reaches 100,000, the prize money of 1,000 ether is transferred to that candidate's Ethereum address. This example highlights some advantages of smart contracts in contrast to classical voting scenarios: (i) contract conditions and vote counts are transparent, (ii) existing votes cannot be manipulated or discarded, and (iii) the prize money will definitely be paid as soon as the condition is reached.

In order to use Ethereum smart contracts in swarm robotics, the target robotic platforms need to meet certain requirements in terms of communication, processing, and storage. The size of one Ethereum transaction is around 150 Bytes. In order to communicate with each other, robots should be able to send and receive some Kilobytes per seconds, otherwise, they may not be able to synchronize their blockchains in an adequate amount of time. During the simulations conducted in our research, the blockchain grew on average to 6.8 MB, a size which could be stored on many state-of-the-art robots in swarm robotics^1^.

## 3. Related Work

This section first discusses consensus achievement in robot swarms (section 3.1), followed by work related to security issues (section 3.2), and concludes by reviewing existing work on blockchain technology used in swarm robotics (section 3.3).

### 3.1. Consensus Achievement

Consensus achievement problems in robot swarms can be divided into *discrete* and *continuous* problems (Valentini et al., 2017). Discrete problems can be formalized as best-of-*n* problems, where the swarm has to agree upon a choice among a finite set of *n* choices. Examples of discrete problems are path selection (Montes de Oca et al., 2011), site selection (Reina et al., 2014), and collective perception (Valentini et al., 2016a). In continuous problems, in contrast, the swarm's goal is to agree upon a choice among an infinite set of continuous choices. Examples of continuous problems are collective motion (Ferrante et al., 2012), spatial aggregation (Soysal and Sahin, 2005), and collective estimation (as studied in this work).

In this work, we study the influence of Byzantine robots on efficiently reaching swarm consensus in a continuous collective estimation problem. However, *exact consensus* in continuous problems is typically unattainable on spatially distributed robot systems (Elhage and Beal, 2010), since it would require each robot to agree upon exactly the same value. Connectivity limitations, large distances, local information, or different sensor readings, can hinder that progress. Although the blockchain can overcome this limitation, for the purpose of comparing our blockchain approach to existing approaches, we here only consider *approximate consensus*. This entails that each robot calculates a weighted local average based on its own estimates and those received from neighbors. A consensus has then been reached as soon as the difference between the maximum and the minimum value in the network is smaller than a given threshold. For the comparison, we selected the commonly used consensus algorithms lcp and w-msr.

#### 3.1.1. Linear Consensus Protocol

The linear consensus protocol (lcp) is the prevailing approach for achieving approximate distributed consensus (Beal, 2016) and has been used in a wide variety of use cases, such as formation control, flocking, and sensor fusion (Olfati-Saber and Murray, 2004; Xiao et al., 2005). The main idea is to reach approximate consensus on a set of beliefs held by the agents.

While this linear consensus protocol achieves high accuracies, it does not account for the presence of Byzantine agents. As a result, a single Byzantine robot keeping a constant value will make all non-Byzantine robots converge to that value (Gupta et al., 2006), potentially fully disrupting the functioning of the robot swarm. This confirms the insights and intuitions presented by Winfield and Nembrini (2006) and Higgins et al. (2009) that fault tolerance in robot swarms cannot be taken for granted and that Byzantine robots can compromise the correct functioning of robot swarms.

#### 3.1.2. W-MSR: Byzantine Approximate Consensus

To overcome the susceptibility to Byzantine interference, LeBlanc et al. (2013) introduced the weighted-mean-subsequence-reduced (w-msr) algorithm as a Byzantine fault-tolerant extension of lcp. w-msr is a state-of-the art method for achieving resilient consensus in distributed sensor networks and robot swarms (Guerrero-Bonilla et al., 2017; Saldaña et al., 2017)

The functioning of w-msr is based on outlier detection: given a design parameter *F*, the algorithm discards the smallest and the largest *F* values received from neighbors, including the agent's own belief. A limitation of the algorithm is that in order to select a proper value for the parameter *F* it assumes that the agents have knowledge of the network topology or that they are able to sustain a desired connectivity through control algorithms, such as flocking (Saulnier et al., 2017). However, this is not always possible in robot swarms since robots might become sparsely connected due to changes in the topology of the network (e.g., due to movements, failing units, or communication problems). As we will show later, W-MSR fails if the number of Byzantine robots is greater than *F* or when confronted with Sybil attacks.

### 3.2. Security Issues in Swarm Robotics

At the outset of swarm robotics research, robot swarms were assumed to be fault-tolerant by design, due to the large number and redundancy of the robot units (Dorigo et al., 2004; Millard et al., 2014). While this assumption holds true in some cases, it has been increasingly called into question when researchers began to study explicit fault detection (Winfield and Nembrini, 2006).

A distinction has been made between endogenous and exogenous fault detection. In endogenous fault detection, robots detect faults in themselves; in exogenous fault detection robots detect faults in other robots (Christensen et al., 2009). In early robotics research, most work was devoted to endogeneous fault detection (see for example, Roumeliotis et al., 1998; Christensen et al., 2008). However, it can be difficult to detect certain endogeneous faults, e.g., a robot might have a broken sensor but only realize it if its sensor readings are compared to its neighbor robots. Therefore, more recently swarm robotics research shifted its focus to exogeneous fault detection. Christensen et al. (2009) present a robot swarm whose robots are programmed to flash their leds in synchrony. led flashing indicates correct functioning of a robot. Therefore, broken robots are easily identified by their non-flashing leds and this identification is made easy by the fact that flashing is synchronized across the robot swarm. A disadvantage of this system is that it can only detect robots that are either completely broken or that report an endogeneous error by not flashing their led anymore: malicious robots cannot be detected nor is exogeneous *partial* fault detection possible. Yet, Winfield and Nembrini (2006) argue that complete failures (e.g., power failure) are significantly less severe than partial failures (e.g., motor failure, communication failure, and sensor failure). One reason for this is that partially failed robots can still unfavorably interact with the remaining robots. For example, because of a broken sensor, they could send wrong sensor readings to other robots, misleading the rest of the swarm. The authors point out that future research should focus on the detection of partial failures; this is what we do in this article.

In the first survey on security issues in robot swarms, Higgins et al. (2009) identify tampered swarm members or failing sensors, attacked or noisy communication channels, and loss of availability as the main threats to robot swarms. Tarapore et al. (2015, 2017, 2019) address the detection of *faulty* robots in both simulated and physical robot swarms. Their method is based on outlier detection using the bioinspired crossregulation model. To this end, robots exchange their behavior vectors. Outliers (faulty robots) are detected by comparing the behavior vectors to other behavior vectors in the swarm: if the majority of the swarm has the same behavior vector, this behavior is classified as an inlier, otherwise as an outlier. While this approach does not require a priori knowledge about abnormal behavior, it assumes that every robot shares its behavior vector truthfully.

Security issues related to external factors, such as attacks on the swarm, only started to be studied recently. For example, Zikratov et al. (2016) propose a reputation-based management system where robots keep trust levels about each other based on the correct execution of a predefined protocol. Sargeant and Tomlinson (2016) study a wider range of attacker strategies, such as eavesdropping, data manipulation, and denial of service in robot swarms. Primiero et al. (2018) show that the propagation of deceitful information through the swarm can be prevented if robots probabilistically change their belief.

In contrast to the systems presented above, the blockchain is capable of logging events in a tamper-proof way and of implementing generic meta-controllers. Moreover, all of the above-mentioned systems are susceptible to attacks: e.g., using the led flashing method of Christensen et al. (2009), an attacker can flash its leds in synchrony but send wrong sensor values to the remaining swarm members. The other systems that rely on wireless messages are susceptible to Sybil attacks: without a trusted third-party, it is always possible for a malicious agent to create an unlimited number of new identities in peer-to-peer networks (Douceur, 2002). Through this large number of identities, an attacker can gain a disproportionate amount of power (Gil et al., 2017), potentially causing much damage, e.g., in voting scenarios. The blockchain can prevent Sybil attacks from disrupting swarm behavior by introducing *scarcity* to decentralized systems: a robot wanting to exert influence must pay for this by spending a scarce resource (cryptotokens). It is thus, not the number of entities forged but rather an attacker's wealth that determines the success of the attack.

### 3.3. Related Work on Blockchain Technology in Robot Swarms

In swarm robotics research, it is often assumed that robots do not have access to shared knowledge. This is mainly due to three reasons: (i) it could be unfeasible to set up the infrastructure for such a shared knowledge system; e.g., if the robots are in a remote area and scattered throughout a large physical space; (ii) the shared knowledge system could represent an unacceptable single point of failure; and (iii) it might be computationally too complex to process all incoming and outgoing data in a single system. However, robot swarms could greatly benefit from shared knowledge, for example, for determining whether a consensus has been reached within the swarm, for calculating the mean value of the sensor readings of the single robots, or for determining malfunctioning units. Hence, decisions could be based on a shared view of the world. This would not only possibly simplify several swarm robotics tasks but also enlarge their field of applications facilitating decision processes.

Castelló Ferrer (2016) was the first to describe a variety of use cases for using a blockchain in robot swarms, such as secure communication, data logging, and consensus agreement. Strobel et al. (2018) delivered the first proof-of-concept, using the blockchain framework Ethereum and the robot swarm simulator ARGoS in a binary collective decision scenario. The authors show how a blockchain-based meta-controller improves the quality of the collected sensor data by providing a blockchain security layer on top of existing algorithms developed by Valentini et al. (2016a). The meta-controller detects inconsistencies in a robot's behavior when it deviates from the agreed-upon behavior and excludes it from the swarm. In contrast, prior collective decision-making algorithms could not reach a consensus whenever one or more robots in the swarm are Byzantine.

Fernandes and Alexandre (2019) and Lopes and Alexandre (2019) study the use of blockchain technology for the registration of robotic events (e.g., robot *x* finished job *y*) in industrial scenarios, where the different robots might come from different manufacturers. The authors additionally demonstrate the use of blockchain-based smart contracts for anomaly detection. However they do not assume local time-delayed communication and maintenance of the blockchain among the robots but rather use the blockchain as an external computing platform. Other work addressed obstacles that might hinder the use of blockchain-based controllers in real-world applications. McAbee et al. (2019) discuss how blockchain technology can help to solve problems in military intelligence applications. Nishida et al. (2018) outline an approach to reduce the blockchain size for information sharing in swarm robotics systems by storing the hash of data—in their case image data—in the blockchain instead of the information itself.

The work presented in this article is based on two previous works (Strobel and Dorigo, 2018; Strobel et al., 2018). However, it is significantly extended: (i) instead of solely determining if there are more black or white tiles (i.e., a binary decision task), in the present work, the swarm's goal is to determine the relative frequency of white tiles expressed as a value between 0.0 and 1.0—a collective *estimation* scenario which yields more information and might be more interesting for real-world deployments; (ii) as soon as a consensus on a specific value is reached, the experiment can be stopped in a fully decentralized way via the consensus mechanism of the blockchain; (iii) in the present article, we study different distributions of the features of the scenarios; (iv) we show how the blockchain limits the number of messages a robot can send, thus preventing Sybil attacks; (v) we present the ARGoS-blockchain interface which enables researchers to test and extend the presented scenarios on different platforms.

## 4. ARGoS-Blockchain Interface

**Figure 4:**
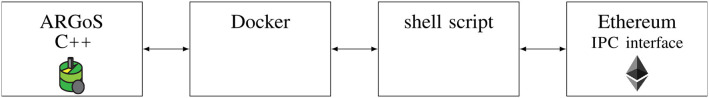
For each robot, the ARGoS-Blockchain interface establishes a connection to the Ethereum blockchain via a Docker container and shell scripts that provide templates for executing Ethereum (geth) functions.

The ARGoS robot simulator (Pinciroli et al., 2012) is the state-of-the-art research platform to conduct simulations in swarm robotics. In our research, each robot acts as an Ethereum blockchain node, maintaining a custom Ethereum network. In order to connect ARGoS and Ethereum, we developed the ARGoS-Blockchain interface that provides access to the Ethereum nodes for the robots (Figure 4). The interface is intended to facilitate research in blockchain-based robot swarms by allowing to call Ethereum functions in ARGoS. Additionally, Docker makes it easy to install and run the interface on different platforms. The interface is available on GitHub^2^.

The implementation of the custom Ethereum network is based on Capgemini aie's Ethereum Docker^3^. Docker containers (Merkel, 2014) contain all the necessary dependencies to run specific applications and are more lightweight than a virtual machine. In our setup, for each robot, the Ethereum implementation *geth* is executed in a separate Docker container. The simulated robots maintain a *custom* Ethereum network, i.e., a network that is shared among the simulated robots and independent of Ethereum's main network. Different containers can communicate with each other via channels.

In order to execute an Ethereum function (e.g., create a new smart contract) from ARGoS, a robot uses its C++ controller to attach to the Docker container. The Docker containers provide shell scripts^4^ with customizable templates (e.g., one of the templates compiles the smart contract, uses the binary code to send a blockchain transactions, and waits until the contract is mined). Via Ethereum's ipc (interprocess communications) interface, the shell scripts execute the Ethereum functions.

**Figure 5:**
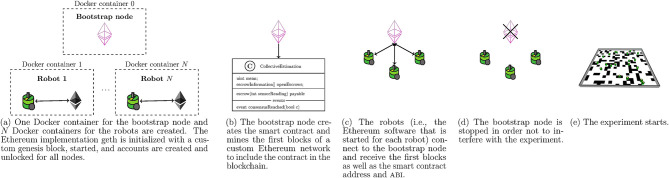
This scheme shows the initialization phase that is executed at the start of each experimental run.

We use an auxiliary “bootstrap” node for publishing the smart contract to the blockchain at the beginning of each run of the simulations (Figure 5). The bootstrap node then mines the smart contract and sends the contract address and the abi (application binary interface; the abi specifies which functions a smart contract provides and how to call them) to the controllers of the robots. As soon as this is done, the bootstrap node is removed from the network. The bootstrap node is not necessarily required and the smart contract could also be created by a robot. However, we used an auxiliary node to make sure (i) that the smart contract is available at the start of the actual experimental run and (ii) that robots have the same initial conditions in all experiments.

The experiments were conducted on a computer cluster. To simulate the limited hardware of real robots, one core with 2.0 GHz and 1.8 GB of memory was assigned to each Docker container^5^. The communication channels between the Docker containers were only established when robots were within a 50 cm communication range in order to simulate the local communication capabilities of real robots.

## 5. Materials and Methods

### 5.1. Setup of the Simulations

We compare three consensus algorithms (lcp, w-msr, and blockchain) in terms of their general performance and resilience to an increasing number of Byzantine robots. To this end, *N* = 20 robots are used in the robot swarm simulator ARGoS (Pinciroli et al., 2012). The swarm's goal is to estimate the relative frequency of white tiles in a 2 × 2 m^2^ “checkerboard” environment where the floor is covered with *B* black and *W* white tiles of size 10 × 10 cm^2^, *B*+*W* = 400 (Figure 1). The checkerboard environment, obstacle avoidance, and random walk movement routines were developed in earlier work by Valentini et al. (2016a). We replicate their parameters for the random walk and obstacle avoidance routines. Depending on the scenario, the positions of the black and white tiles are either fixed by the experimenter or selected randomly at the beginning of a simulation run. The starting positions of the robots are randomly chosen from a uniform distribution at the beginning of each simulation run. To enable the swarm to aggregate information about the environment, each robot samples its local ground sensor and exchanges information with other robots in their communication range. The experiment is conducted in discrete time steps with one time step corresponding to 1 s. At each time step, a robot *i* determines if it is above a black or a white tile via its ground sensor. Each robot works in exploration *phases*. We use the subscript notation _*i, m*_ for variables referring to a robot *i* in its *m*th exploration phase. The duration of each exploration phase is *d* = 45 s. To obtain a sensor reading, a robot *i* in its *m*th exploration phase calculates the ratio ${\hat{\rho }}_{i,m}^{\prime }$ between the number of white tiles Ŵ_*i, m*_ and the total amount of tiles ${Ŵ}_{i,m}+{\hat{B}}_{i,m}$ it sensed in this exploration phase: ${\hat{\rho }}_{i,m}^{\prime }=\frac{{Ŵ}_{i,m}}{{Ŵ}_{i,m}+{\hat{B}}_{i,m}}\in \left[0,1\right]$ . If the distance between two robots is <50 cm, they are in communication range and can exchange information, in accordance with real swarm robotics systems that have only local communication capabilities. This communication range leads to an average degree of connectivity of 2.4 (i.e., one robot is, on average, connected to 2.4 other robots) and yields multiple non-connected clusters that exist almost all the time. For the different approaches, 40 simulation runs (i.e., repetitions) were performed for each value of the independent variable. These are the common characteristics for all three consensus protocols. The peculiarities of the different consensus protocols are given in section 5.2.

### 5.2. Implementation of the Different Consensus Models

#### 5.2.1. Linear Consensus Protocol

**Figure 6:**
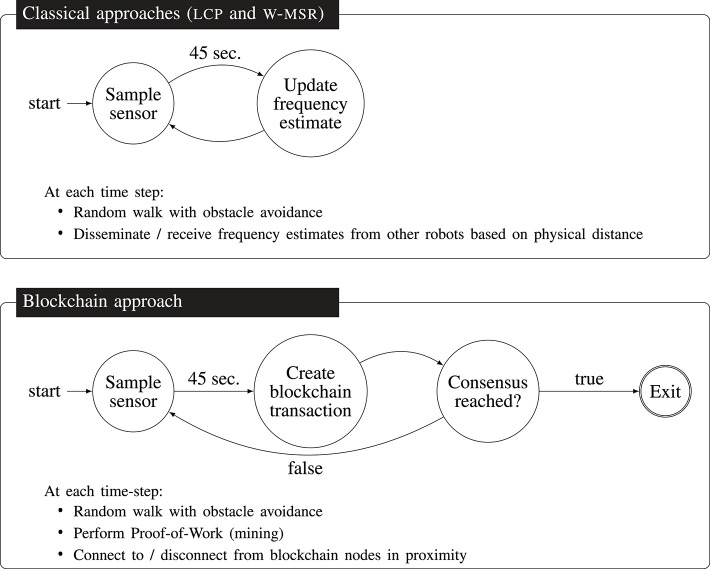
The robots explore the environment using a random walk routine and sample their ground sensors. Using the classical approaches lcp and w-msr **(top)**, they update their frequency estimate every 45 s (i.e., every 45 time-steps) via Equation (1). Using the blockchain approach **(bottom)**, every 45 s, the robots create a blockchain transaction that includes their sensor reading. In contrast to the classical approaches, with the blockchain approach, the robots can check whether a consensus has been reached by querying the state (true or false) of the smart contract event consensusReached. If true, they enter the exit state and stop creating blockchain transactions. Then, they still perform the random walk and connect to other robots in their proximity to exchange blockchain information.

Using the linear consensus protocol (lcp), each robot keeps track of a frequency estimate ${\hat{\rho }}_{i,m}$ that represents its belief about the relative frequency of white tiles. At the end of the first exploration phase (*m* = 0), the frequency estimate is set to the sensor reading of the first phase: ${\hat{\rho }}_{i,0}={\hat{\rho }}_{i,0}^{\prime }$. The frequency estimate is then updated at the end of each 45 s exploration phase *m* by incorporating the frequency estimates ${\hat{\rho }}_{j,m-1}$ of the neighbors $N_{i}$ (Figure 6):

(1) $$\begin{array}{r} {\hat{\rho }}_{i,m}=w_{ii}{\hat{\rho }}_{i,m}^{\prime }+\underset{j\in N_{i}}{\sum }w_{ij}{\hat{\rho }}_{j,m-1}, \end{array}$$

where $w_{ii}=w_{ij}=\frac{1}{|N_{i}|+1}$ is a weight factor, assigning each message an equal weight, as done in related work (e.g., Saulnier et al., 2017). In the phase *m*+1, a robot *i* distributes its frequency estimate ${\hat{\rho }}_{i,m}$ to other robots in communication range, i.e., robots communicate their frequency estimates and not their current sensor readings (the sensor readings fluctuate from phase to phase and consensus achievement would be difficult if these values were used). As in the work by Valentini et al. (2016a), each robot has an identifier and only one message can be received from any specific robot in each phase. In order to store received messages, robots have a buffer size of *M* = *N*−1 = 19. If more messages are received, only the last *M* messages are stored. The buffer size *M* = 19 makes sure that every robot is able to receive a message from every other robot in each exploration phase but small enough so that it represents a mechanism to prevent flooding of the network.

#### 5.2.2. W-MSR

The w-msr algorithm is a variant of lcp and introduces a means for detecting and discarding outliers. It also uses Equation (1) to obtain a consensus but first performs outlier detection. To do so, the outliers are removed from the set of neighbors. The algorithm requires a design parameter *F* that should be selected based on the assumed number of Byzantine robots and connectivity of the network. We set *F* = 2. Then, all received values ${\hat{\rho }}_{j,m-1}$ larger than ${\hat{\rho }}_{i,m}^{\prime }$ are sorted in ascending order. If there are fewer than *F* values larger than ${\hat{\rho }}_{i,m}$, all of them are added to the set of outliers O. Otherwise, the *F* largest values are considered outliers. The same procedure is applied to all values smaller than ${\hat{\rho }}_{i,m}^{\prime }$. To update the frequency estimate, the w-msr algorithm then uses $N^{\prime }=N\backslash O$ instead of N in Equation (1).

#### 5.2.3. Blockchain Approach

**Figure 7:**
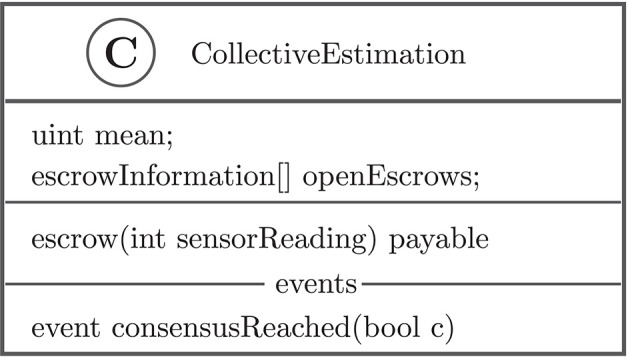
The smart contract keeps track of the frequency estimate ${\hat{\rho }}_{t}$ and provides the function escrow to send a sensor reading ${\hat{\rho }}_{i,m}^{\prime }$. The event consensusReached is set to true when the frequency estimate does not change more than τ from one escrow round to the next one.

The blockchain approach is based on a smart contract that aggregates the sensor readings of the robots into the frequency estimate ${\hat{\rho }}_{t}$, while discarding outliers and rewarding robots for contributing to the scenario (Figure 7). To be consistent with the classical approaches, we will use the notation ${\hat{\rho }}_{i,m}$ to indicate the estimated frequency of white tiles as written in the blockchain of robot *i* in its *m*th exploration phase, but will otherwise write ${\hat{\rho }}_{t}$ to indicate the frequency estimate in escrow round *t* (see below for a description of the escrow).

Using the blockchain approach, the robots' sensor information is stored and aggregated using a smart contract at given time intervals (Figure 6). Each robot keeps a local copy of the blockchain; if robots are physically close to each other, they exchange their blockchain information. The setup uses the ARGoS-Blockchain interface described in section 4. In order to simulate the local communication capabilities of real robots, the simulated robots have the ability to connect to each other's Ethereum processes via the Docker container if their distance is smaller than 50 cm; they can then exchange blocks and unconfirmed transactions of the blockchain. To synchronize ARGoS and Ethereum, the experiments were conducted in real time.

Each robot *mines*, i.e., it performs the Proof-of-Work, from the start to the end of a simulation run. Every time a robot successfully solves a block, it is rewarded by 5 ether (Ethereum's cryptocurrency^6^). In the beginning of each experimental run, all robots have a balance of 0 ether. Since creating blockchain transactions requires ether, robots have to mine blocks to gain ether and be able to send transactions to the smart contract. The robots start with 0 ether so that we do not need to identify beforehand which robots will be part of the experiment. This builds a basis for “open robot swarms” (e.g., for citizen science projects) where robots are free to join and leave at any time during an experiment.

We specified an initial difficulty of the mining puzzle in the genesis block, so that the swarm mines approximately one block per second, resulting in 2.25 blocks per robot after 45 s. Therefore, the average balance after 45 s is 2.25 × 5 ether = 11.25 ether. This means that after 45 s most of the time none of the robots have enough ether to submit a transaction. Note that it is possible for the robots to mine empty blocks, i.e., blocks without any transactions, and still get the reward of 5 ether for solving the block.

**Figure 8:**
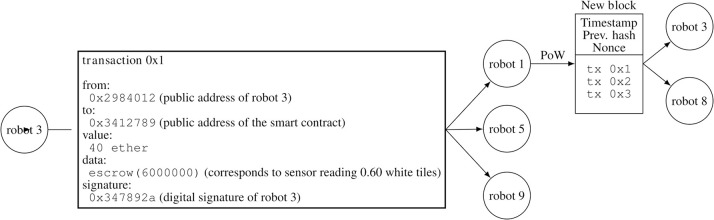
In this example, robot 3 creates the transaction tx 0x1 in order to send its sensor reading to the smart contract. The transaction is then disseminated to its neighboring robots 1, 5, and 9. Since the transaction is not included in a block yet, it is an unconfirmed transaction. Robot 1 is able to mine a new block that includes this transaction and two other transactions tx 0x2 and tx 0x3. Robot 1 then disseminates this mined block among its neighboring robots 3 and 8.

At the end of each exploration phase *m* (i.e., after 45 s), each robot sends its sensor reading ${\hat{\rho }}_{i,m}^{\prime }$ to the smart contract via the function escrow(int sensorReading) (Figure 7) and the value gets stored in the list openEscrows. That is, to store a sensor reading in the blockchain (Figure 8), a robot (i) creates a blockchain transaction which includes its sensor reading in the data part of the transaction, (ii) adds a deposit amount of *q* ether, (iii) signs this transaction, and (iv) disseminates this transaction among its neighboring robots. The function escrow accepts a value between 0.0 and 1.0, which stands for the sensor reading ${\hat{\rho }}_{i,m}^{\prime }$ of the robot *i*. Since smart contracts in Ethereum accept integer values only, in the actual implementation, all sensor readings are multiplied by 10^7^ to simulate rational numbers between 0.0 and 1.0 (e.g., instead of sending 0.30, a robot would send 0.30 × 10^7^). The deposit amount *q* is intended to limit the number of sensor readings a robot can send. That is, a robot “vouches” for its sensor reading. When the robots send transactions, they do not check whether they possess enough ether or not: in case they do not have enough ether, the transaction is simply discarded by the smart contract. We set *q* = 40 ether, a suitable value as determined in a pilot experiment.^7^

The goal of the escrow is to collect sensor readings and to reward robots that sent meaningful sensor data. As soon as the length of openEscrows is equal to *V* = 20, a new disbursement round *t* is performed, i.e., outliers are identified and inliers are rewarded. To this end, the difference between ${\hat{\rho }}_{t}$ (frequency estimate in the smart contract in disbursement round *t*) and ${\hat{\rho }}_{i,m}^{\prime }$ (sensor readings from the individual escrow transactions) is determined. If the absolute difference $\left|{\hat{\rho }}_{i,m}^{\prime }-{\hat{\rho }}_{t}\right|$ is smaller than a threshold ϵ, the sensor reading is accepted, otherwise it is discarded. Accepted values of ${\hat{\rho }}_{i,m}^{\prime }$ are called inliers, discarded ones are called outliers. The value of the mean ${\hat{\rho }}_{t}$ is obtained by calculating the mean of all inliers over all escrow rounds *t*. In every new escrow round, it is updated via a one-pass algorithm to reduce the computational requirements. The execution of the smart contract includes activities such as verifying the validity of the transaction and, when it has received *V* = 20 valid transactions, to compute the mean. The smart contract is executed every time a block is mined (as long as it includes transactions) but the computation of ${\hat{\rho }}_{t}$ usually happens with a lower frequency.

In the first round (*t* = 0)—i.e., in the time interval from the beginning of the experiment to the moment in which the smart contract has received 20 valid transactions—when no frequency estimate ${\hat{\rho }}_{t}$ is available yet, all values of ${\hat{\rho }}_{i,m}^{\prime }$ are accepted. The value of ϵ is a tuning parameter that influences how much the current mean in the blockchain can change from one round to the other. Decreasing ϵ will increase the sensitivity (the number of Byzantine votes that are correctly identified as outliers), while increasing ϵ will increase the specificity (the number of non-Byzantine votes that are correctly included in the calculation of the current mean). We set ϵ = 0.2, a suitable value as determined in a pilot experiment. The value *V* is another tuning parameter: lower values lead to earlier results for ${\hat{\rho }}_{t}$ (since the value is only updated at the end of an escrow round) but also to an increased risk that the ratio between the number of Byzantine robots and normal robots is high in an escrow round. If the value of *V* is set too high, the detection of Byzantine robots may start too late and they might have already caused a significant damage and non-Byzantine robots may have to wait long until they get back their deposit amount. We set the list length to *V* = 20 = *N* since then, on average, every robot will be represented by one vote in each round.

In order to incentivize robots to take part in the escrow, inliers get a reward *r*_*t*_ in ether. The reward *r*_*t*_ is greater or equal to the escrow value and calculated by distributing the collected ether of the escrow round among the inliers: *r*_*t*_ = *Vq*/*in*_*t*_ = 20 × 40 ether/*in*_*t*_, where *in*_*t*_ is the number of inliers at round *t*. Hence, robots can gain ether by mining, thereby improving the network's security, or by sending sensible sensor values, helping to determine the correct frequency of white tiles. This creates an implicit reward mechanism within the swarm that discourages Byzantine robots to operate as such, since sending wrong sensor measures costs cryptotokens.

### 5.3. Software Availability

The implementation of the presented classical approaches^8^ and blockchain approach^9^ are hosted on GitHub.

### 5.4. Statistics

Let R=1,2,…,N be the set of all robots, G be the subset of non-Byzantine robots (mnemonic: G for “good”) and B be the subset of Byzantine robots (mnemonic: B for Byzantine or “bad”), with G∪B=R and |B|=k. An asterisk * indicates a randomly selected robot from the set R and the infinity symbol ∞ indicates that the value is determined at the end of an experimental run. Therefore, ${\hat{\rho }}_{*,\infty }$ is the estimated frequency of white tiles of one randomly selected robot at the end of an experimental run and ${\hat{\rho }}_{G,\infty }=\underset{i\in G}{\sum }{\hat{\rho }}_{i,m}/|G|$ is the arithmetic mean of the frequency estimate of all non-Byzantine robots at the end of an experiment. The frequency estimate ${\hat{\rho }}_{B_{0},\infty }$ indicates the mean of the frequency estimate of a run where the number of Byzantine robots was zero ($B_{0}$). We use the median ${\tilde{\hat{\rho }}}_{B_{0},\infty }$ as a baseline value to compare the performance of the approaches, when the number of Byzantine robots is increased. The baseline values are determined separately for the lcp, w-msr, and blockchain approaches.

The following statistics are used to compare the performances of the three approaches:

- **Absolute error** ae_*_. This statistic is the absolute value of the difference between the actual relative frequency of white tiles ρ and the frequency estimate ${\hat{\rho }}_{*,\infty }$ of a randomly selected robot at the end of an experimental run: ${\text{AE}}_{*}=|\rho -{\hat{\rho }}_{*,\infty }|$. ae_*_ measures the predictive capacity of the different approaches. For the calculation of ae_*_, we randomly select one robot since we assume that a consensus has been reached. Compared to averaging the values of all or several robots, this approach is closer to real-world scenarios where only a single functioning robot might be retrieved after the end of an experiment; additionally, it might be too time-consuming or costly to sample all the robots.
- **Harm**. This statistic measures the amount of harm that Byzantine robots cause to non-Byzantine robots. The idea is that we compute the difference between (i) ${\text{AE}}_{G}$, that is, the average absolute error of the non-Byzantine robots in presence of Byzantine robots, and (ii) ${\tilde{\text{AE}}}_{B_{0}}$, that is, the median of the average absolute error over all runs with zero Byzantine robots: $\text{harm}={\text{AE}}_{G}-{\tilde{\text{AE}}}_{B_{0}}$. Note that to calculate the harm we take on an “omniscient perspective” and assume that we are able to distinguish between Byzantine and non-Byzantine robots. That is, for analysis purposes, here we consider the case when it is possible to retrieve all the robots and identify those that are non-Byzantine after the experiment.
- **Consensus time** ***T*_*N*_**. This statistic is the time in seconds until all robots have reached a consensus on a certain estimated frequency of white tiles (see section 6.2.2).

For the blockchain approach, additionally, the following statistic is measured:

- **Blockchain size** **BC_MB_**. This statistic indicates the blockchain size in MB of one randomly chosen robot, determined at the end of each experimental run.

For all plots showing the absolute error ae_*_ and the Harm in the presence of Byzantine robots, we additionally perform locally estimated scatterplot smoothing (loess^10^) indicated by blue curves in the graphs. The gray bands around the blue loess curve indicate the 95% confidence interval for predictions from the regression. The loess curve is intended to make it easier to spot the general trend when the number of Byzantine robots is increased.

## 6. Simulations

In this section, we compare the three approaches (lcp, w-msr, blockchain) in five experiments under different conditions (Table 1). The experiments are structured along the three research questions introduced in section 1 and correspond to the complexity and intelligence of Byzantine robots.

**Table 1:** Overview of the experiments.

**No**.	**Experiment**	**% White tiles**	**# Byzantines**	**Tile mixing**	**Sybil attack**	**Exit criterion**
1	Random distribution (no Byzantines)	0, 10, …, 100	0	Yes	No	1, 000 s
2	Random distribution	75	0, 1, …, 7	Yes	No	1, 000 s
3	Consensus	75	0, 1, …, 7	Yes	No	Threshold below τ
4	Binary distribution	75	0, 1, …, 7	No	No	1, 000 s
5	Sybil attack	75	0, 1, …, 7	Yes	Yes	1, 000 s

- **Baseline**: Experiment 1 is intended to establish a baseline and does not contain any Byzantine robots. It tests the three different approaches in an environment with randomly distributed tiles. The goal of the experiment is to provide a proof-of-concept and show that the approaches work as intended in standard conditions.
- **Byzantine Robots:** Experiments 2–4 introduce Byzantine robots. While there are many possible Byzantine failures, in this work we study a case where each Byzantine robot disseminates a frequency estimate of ${\hat{\rho }}_{i,m}=0.0$ for the classical approaches and accordingly ${\hat{\rho }}_{i,m}^{\prime }=0.0$ for the blockchain approach in all exploration phases *m*, independent of its actual sensor readings. This choice is motivated by two reasons: (1) a value of 0.0 is the worst-case scenario and maximizes the difference between ρ and ${\hat{\rho }}_{i,m}$ and (2) it is a failure mode studied in other research (e.g., Gupta et al., 2006). We vary the number of Byzantine robots between 0 and 7.^11^
- **Sybil attack:** Experiment 5 then introduces clearly malicious Byzantine robots that perform Sybil attacks. The malicious robots still disseminate frequency estimates of ${\hat{\rho }}_{i,m}=0.0$ and ${\hat{\rho }}_{i,m}^{\prime }=0.0$. However, they try to send as many messages as possible by creating new identities at every time step. The goal of this experiment is to show that just one malicious robot suffices to let existing approaches fail.

### 6.1. Comparison in Absence of Byzantine Robots

In the first experiment, we compare the values of ae_*_ for the different approaches without the presence of Byzantine robots. To this end, the percentage of white tiles is increased from 0 to 100% in steps of 10%. A simulation run is stopped after 1,000 seconds. The goal of this experiment is to (1) determine if the blockchain-based approach can replace existing approaches, (2) establish a baseline for successive experiments, and (3) see if all approaches are able to deal with a straightforward experimental setup.

#### Results, Discussion, and Interpretation

**Figure 9:**
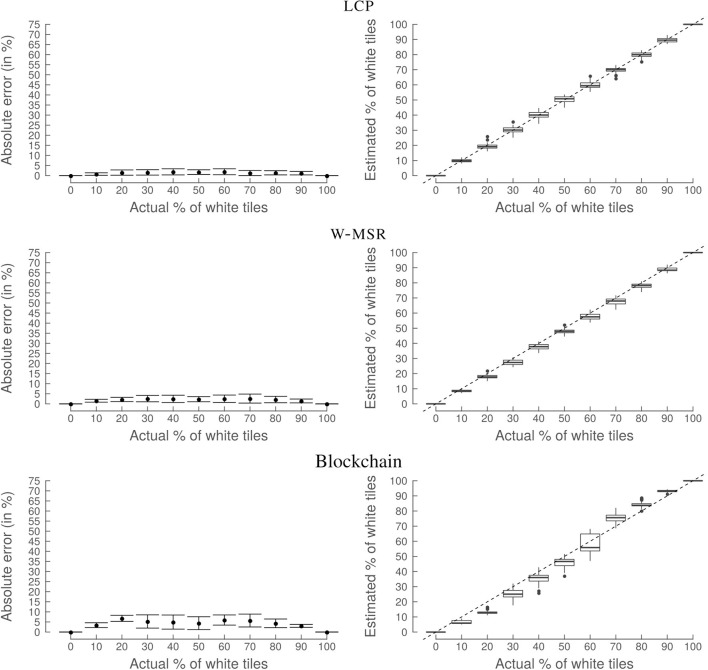
Experiment 1: Random distribution (no Byzantines). lcp **(top)**, w-msr **(middle)**, and the blockchain approach **(bottom)** perform well if the tiles are randomly distributed and if there are no Byzantine robots. This result serves as a baseline for the following simulations. No correlation between the actual frequency of white tiles and the absolute error (ae_*_) is visible. The graphs on the left-hand side show the mean with the error bars indicating the standard deviation. The dashed line in the plots on the right-hand side show the ideal outcome, i.e., when the true % of white tiles equals the estimated % of white tiles.

The three approaches perform well with a mean absolute error lower than 0.08 (Figure 9) and are, therefore, able to successfully perform the desired task. However, the blockchain approach presents a slightly higher variability and mean absolute error for some values of the actual % of white tiles. This is because the blockchain approach has more random factors—due to the Proof-of-Work and the specific security measure implemented in the smart contract—than the classical approaches. The overall good performance serves as a baseline for the following scenarios.

### 6.2. Comparison in Presence of Byzantine Robots

In the next three simulations, we study the influence of Byzantine robots (robots that disseminate ${\hat{\rho }}_{i,m}=0.0$ for the classical approaches and ${\hat{\rho }}_{i,m}^{\prime }=0.0$ for the blockchain approach) on the performance of the different approaches. The number of Byzantine robots is increased from 0 to 7. The frequency of white tiles in the environment is fixed to 75 %. We chose 75 % because it is in the middle between 50 % and 100 %, i.e., it contains a bias for one color to rule out that a random approach might work.

#### 6.2.1. Byzantine Robots in a Random Environment

In this experiment, the influence of Byzantine robots on the value of ae_*_ is studied in an environment with randomly distributed tiles. A simulation run is stopped after 1,000 s. The goal of this experiment is to investigate how the Byzantine robots affect the different approaches. With an increasing number of Byzantine robots, we expect lcp to break down fairly quickly due to its lack of security measures. In contrast w-msr and the blockchain approach should be more resilient as long as the number of Byzantine robots remains low.

##### Results, discussion, and interpretation

**Figure 10:**
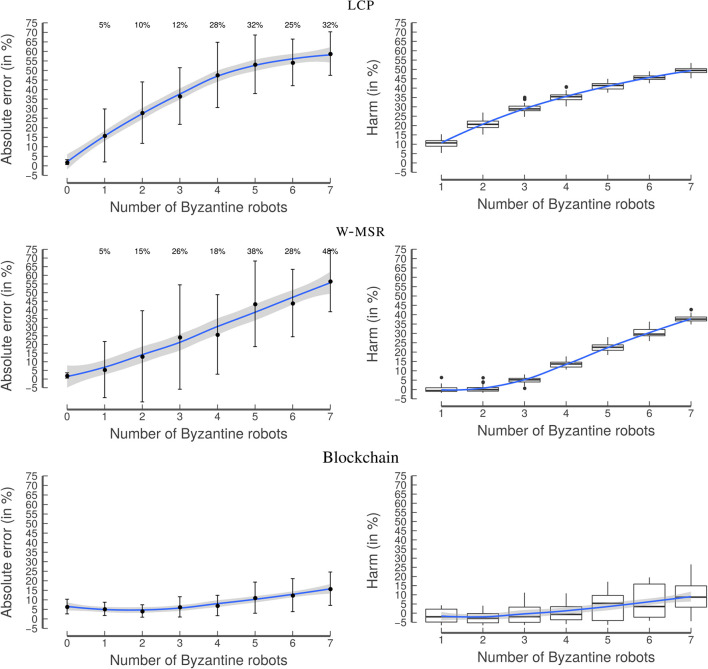
Experiment 2: Random distribution. When the number of Byzantine robots increases, lcp's performance **(top)** quickly deteriorates and the frequency of extreme outliers becomes high (the percentages at the top of each graph correspond to the frequency with which Byzantine robots were selected when calculating ae_*_). Therefore, with the classical approaches, one is always exposed to the risk of getting a completely wrong result, even if there is just one Byzantine robot in the swarm. w-msr **(middle)** is able to manage a few Byzantine robots but its ae_*_ quickly increases when there are more than three of them. The blockchain approach **(bottom)** is largely unaffected by the increasing number of Byzantine robots, and does not contain extreme outliers. The graphs on the left-hand side show the mean with the error bars indicating the standard deviation. The blue line is obtained by locally estimated scatterplot smoothing (loess), the gray band around the blue line shows the 95% confidence interval for predictions from the loess regression.

The lcp approach is not designed to be resilient to the presence of Byzantine robots; accordingly, a strong increase in its ae_*_ can be observed when the number of Byzantine robots increases (Figure 10). In contrast, by design, w-msr is resilient to the presence of Byzantine robots, as long as their number is low. However, both approaches have a high standard deviation, partially due to the high number of extreme outliers where the ae_*_ is 75%. This is due to the fact that ae_*_ is computed by randomly selecting a robot from the swarm. When the number of Byzantine robots increases the probability of selecting a Byzantine robot increases. While different choices of w-msr's design parameter *F* would lead to different values for ae_*_, the percentage of extreme outliers would stay the same (since the Byzantine robots do not follow the protocol); additionally, in a real-world scenario one would not be able to know whether the selected robot is Byzantine or not.

The blockchain approach is resilient also to a higher number of Byzantine robots. In contrast to the classical approaches, even if a Byzantine robot is selected, the ae_*_ stays low. This is due to the consensus protocol of the blockchain, i.e., all robots agree on the longest chain and even the Byzantine robots share the same estimate written in the blockchain.

Particularly interesting is the harm value of the lcp. It starts with a median of more than 10% for one Byzantine robot. In other words, the estimated frequency of all non-Byzantine robots is already 10% worse compared to the baseline, if just 5% of the robots (1 out of 20) are Byzantine. The harm can also be negative, in cases when the Byzantine robots help to get closer to the actual ρ. This is the case for the blockchain approach. Without Byzantine robots, the blockchain approach overestimates the frequency of white tiles due to the implemented security measure: since the smart contract only accepts values within ${\hat{\rho }}_{t}-\epsilon <{\hat{\rho }}_{i,m}^{\prime }<{\hat{\rho }}_{t}+\epsilon$, several ${\hat{\rho }}_{i,m}^{\prime }$ values from non-Byzantine robots will be discarded. Therefore, the addition of a small number of Byzantine robots reduces the absolute error and the harm. This is a characteristic of the specific smart contract and different values of ϵ or a different outlier detection method (e.g., taking the standard deviation into account) would lead to different results.

#### 6.2.2. Consensus Agreement in the Presence of Byzantine Robots

In this experiment, the influence of Byzantine robots on the swarm's ability to reach a consensus is studied. The goal of this experiment is to investigate if a swarm can reach a consensus in a fully decentralized way.

For the classical approaches (lcp and w-msr), we say that a consensus in the swarm has been reached once the absolute difference between the highest ${\hat{\rho }}_{i,m}$ and the lowest ${\hat{\rho }}_{j,m}$ in the swarm is smaller than a threshold value τ. However, as soon as there is one “stubborn” Byzantine robot that keeps a constant frequency estimate, consensus of all robots can only be on that value when using the classical approaches. In our case, if the robots would come to a consensus, the only possible value would be 0.0, therefore, the expected absolute error would be 75% for the classical approaches, resulting in a useless frequency estimate of the swarm. For this reason, we show the consensus time for the classical approaches only in the *absence* of Byzantine robots.

For the blockchain approach, consensus is reached, if the frequency estimate between two escrow rounds does not change more than τ, i.e., $|{\hat{\rho }}_{t}-{\hat{\rho }}_{t-1}|<\tau$. The blockchain event consensusReached is then set to true. At the end of each exploration phase, each robot queries the status of this event. If the status of the event is true for all robots, the simulation run is stopped. For this experiment, we use the consensus threshold τ = 0.02.

##### Results, discussion, and interpretation

**Figure 11:**
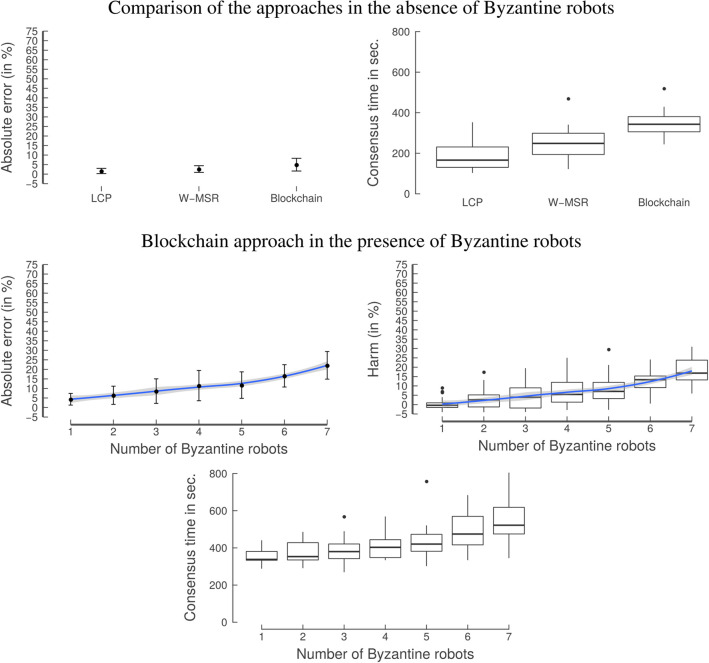
Experiment 3: Consensus. **(Top)** In the absence of Byzantine robots all approaches are able to reach a consensus in a reasonably short amount of time. However, there is a trade-off between consensus time in the absence of Byzantine robots and the level of security an approach provides. **(Bottom)** When testing the swarm's ability to reach a consensus, the classical approaches can only reach a consensus on the values of the Byzantine robots. In contrast, the blockchain approach continues to work. It shows a slight increase in the consensus time when the number of Byzantine robots is increased; this happens due to the increased variance that is introduced by the increasing number of Byzantine robots. The graphs on the left-hand side show the mean with the error bars indicating the standard deviation. The blue line is obtained by locally estimated scatterplot smoothing (loess), the gray band around the blue line shows the 95% confidence interval for predictions from the loess regression.

The top row in Figure 11 shows the comparison of the three approaches in the absence of Byzantine robots. All approaches perform well and are able to reach a consensus in a reasonably short amount of time. The consensus time of the w-msr and blockchain approaches is higher than the baseline lcp approach. Hence, there is a trade-off between consensus time in the absence of Byzantine robots and the level of security an approach provides.

The bottom row in Figure 11 shows the absolute error and consensus time of the blockchain approach. The consensus time rises slightly when the number of Byzantine robots increases. Similarly, the absolute error also increases, but the mean of the ae_*_ remains at about 20% even with seven Byzantine robots.

The blockchain-controlled swarm could reach a decentralized consensus, even in the presence of Byzantine robots. Therefore, it is autonomous and resilient, while the classical approaches are not. In addition—even without Byzantine robots—it is difficult for the classical approaches to determine whether each robot actually agrees on a certain value. Note that the classical approaches could be extended, so that robots in the swarm send a consensus signal to their neighbors when they have reached convergence; however, this signal would be prone to Byzantine robots sending a negative consensus signal. In practice, an external observer might be needed but this observer would represent a single point of failure and in some cases it might even be impossible to set it up. In contrast, in the case of the blockchain approach, the consensus determination is done *on-chain* (i.e., via a blockchain-based smart contract) without any external observer.

#### 6.2.3. Byzantine Robots in a Binary Environment

**Figure 12:**
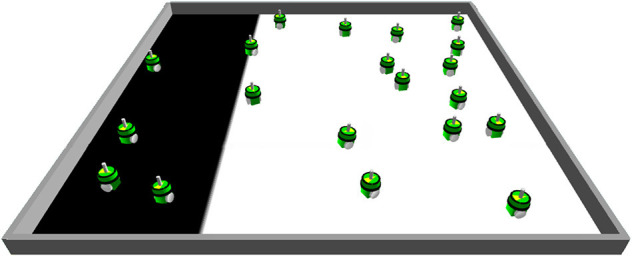
When using the fixed distribution of the tiles, the left part of the environment is covered with black tiles while the rest is covered with white tiles. This fixed distribution is expected to make it more difficult for the smart contract to detect Byzantine robots since normal robots might send the same sensor values as Byzantine robots.

In this experiment, the influence of Byzantine robots on the value of ae_*_ is studied in an environment with a fixed distribution of tiles (Figure 12). Using the fixed distribution, the tiles in the left part of the environment are black (25%), while those in the right part are white (75%). A simulation run is stopped after 1,000 s. The goal of this experiment is to investigate whether the modified distribution of tiles makes the detection of outliers more difficult since also non-Byzantine robots will get extreme sensor readings of ${\hat{\rho }}_{i,m}^{\prime }=0.0$ and ${\hat{\rho }}_{i,m}^{\prime }=1.0$.

##### Results, Discussion, and Interpretation

**Figure 13:**
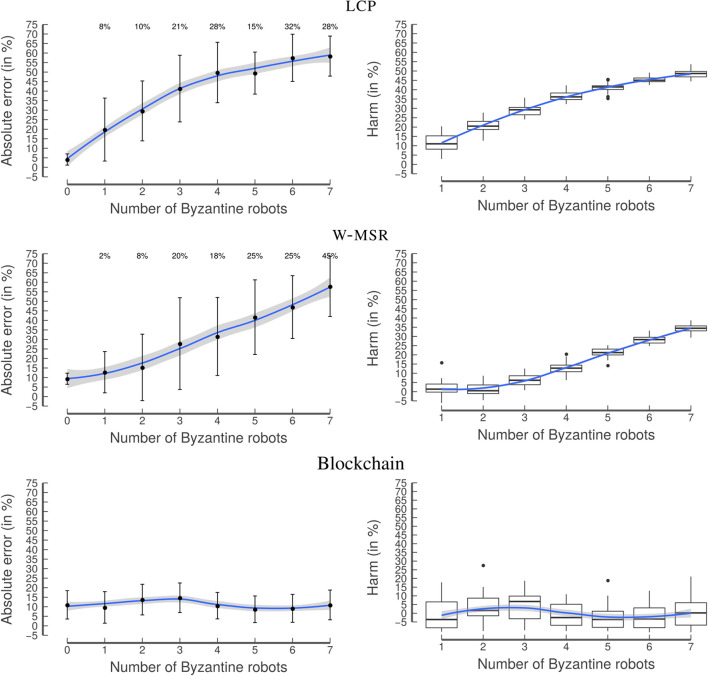
Experiment 4: Binary distribution. Similar to the random distribution, lcp **(top)** shows a steep increase in ae_*_, w-msr **(middle)** can handle a few Byzantine robots, and the blockchain approach **(bottom)** is also resilient to a higher number of Byzantine robots. The graphs on the left-hand side show the mean with the error bars indicating the standard deviation. The blue line is obtained by locally estimated scatterplot smoothing (loess), the gray band around the blue line shows the 95% confidence interval for predictions from the loess regression.

While lcp's ae_*_ quickly increases with an increasing number of Byzantine robots, the w-msr approach is able to manage a few Byzantine robots, starting with a relatively high ae_*_ of 10% (Figure 13).

When no Byzantine robots are part of the swarm, lcp performs better than w-msr and the blockchain approach. This is because of the security measures implemented in w-msr and in the blockchain approach, which have difficulties in distinguishing between the values generated by the Byzantine and by the non-Byzantine robots. However, in contrast to w-msr, the blockchain's performance remains approximately constant, even for a rather high number of Byzantine robots. The harm distribution is similar to Experiment 2.

These results show that there is no “one size fits all” of consensus protocols; instead, there is a trade-off between adding security measures to approaches and their ability to perform well under all circumstances. However, in real-world scenarios, we will almost certainly have to deal with Byzantine robots, therefore, using the blockchain approach is still warranted.

### 6.3. Comparison in Presence of Sybil Attacks

In the last experiment, we study the case in which Byzantine robots perform a Sybil attack. The goal of this experiment is to investigate how decentralized swarms can deal with robots that forge multiple identities. The tiles are randomly distributed and a simulation run is stopped after 1,000 s.

To perform a Sybil attack, the Byzantine robots are programmed as follows. In the classical approaches, every Byzantine robot creates a new identity at every time step and uses it to disseminate its sensor readings. In the blockchain approach, robots do not create new identities since these identities would not have any ether; therefore, the Sybil attack would be prevented automatically. We could have programmed Byzantine robots to first create new public addresses (i.e., identities) and distribute their ether among these addresses but since the public addresses are not used in the identification of outliers, this was not deemed necessary. Additionally, this would most likely weaken the Sybil attack, since first distributing the tokens would slow down the process. Instead, in the blockchain approach, a Byzantine robot sends as many transactions as possible. However, the limiting factor is that sending transactions costs cryptotokens, that is, robots have to send 40 ether every time they send an escrow transaction that contains their sensor reading (section 5.2.3).

#### Results, Discussion, and Interpretation

**Figure 14:**
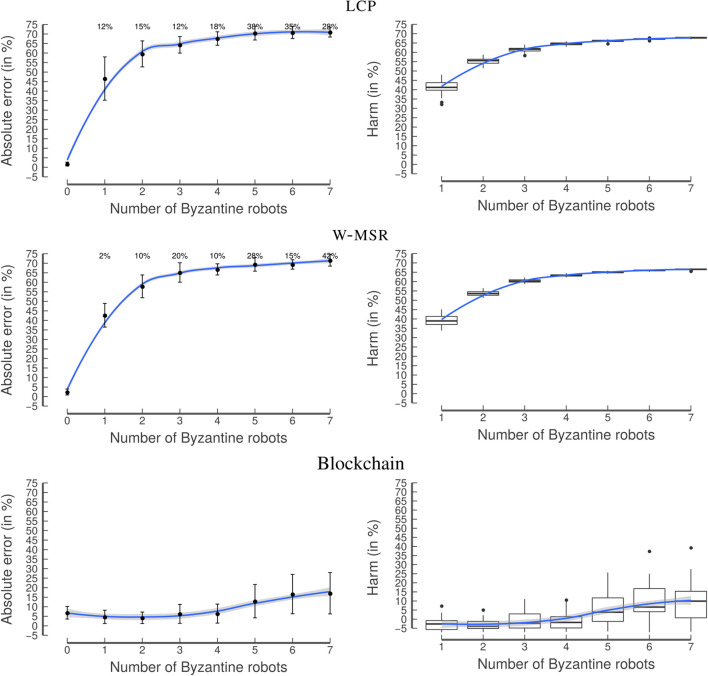
Experiment 5: Sybil attack. In the comparison of the approaches, it is clearly visible that lcp **(top)** and w-msr **(middle)** fail in the presence of even one Byzantine robot performing a Sybil attack. In contrast, the blockchain approach **(bottom)** is able to prevent these attacks by limiting the number of transactions that can be included in the blockchain. The graphs on the left-hand side show the mean with the error bars indicating the standard deviation. The blue line is obtained by locally estimated scatterplot smoothing (loess), the gray band around the blue line shows the 95% confidence interval for predictions from the loess regression.

As expected, the classical approaches have high values for ae_*_ and harm as soon as one robot in the swarm is able to perform a Sybil attack (Figure 14). In stark contrast, in the blockchain approach, the Sybil attack is not successful since the 40 ether robots have to deposit to send a transaction prevents the robots from creating a high number of transactions. In other words, the robots cannot “spam” or “flood” the network with transactions since they would quickly run out of ether. The robots also cannot steal the identity of other robots (spoofing attack) due to digital signatures. Therefore, the blockchain approach stays resilient, even in the presence of a relatively high number of Byzantine robots. Based on these results, one of the main advantages of this approach is visible: the blockchain is able to introduce *scarcity* into a decentralized swarm, making the system more secure.

## 7. General Discussion

In this work, we set out to study whether robot swarms need blockchain technology. To this end, we considered the open research problem of consensus reaching in robot swarms for the general case of Byzantine robots and the more specific case of Sybil attacks. To answer the three research questions listed in the introduction of this article, we used a collective estimation task and compared the blockchain approach to existing consensus protocols. Our simulation results support a positive answer to our research questions: in the absence of Byzantine robots, consensus could be reached as effectively with blockchain-based smart contracts as with existing consensus protocols in robot swarms (RQ 1); the use of smart contracts indeed mitigates the influence of Byzantine robots in robot swarms (RQ 2); and Sybil attacks were prevented when using the blockchain approach (RQ 3). Below, we discuss the implications and limitations of our research.

### 7.1. Implications

The results of our experiments can be generalized in two ways: across use cases and across platforms. We showed that it is possible to implement meta-controllers with blockchain-based smart contracts. In our experiments, a meta-controller (i) aggregated the sensor readings from the individual robots, (ii) performed simple, yet effective outlier detection to manage Byzantine robots, and (iii) determined if a consensus was reached in the swarm, even in the presence of Byzantine robots.

The provided use case was intended to be a simple and easy to understand example of how a smart contract can be used in swarm robotics. Therefore, we used one of the simplest outlier detection methods. As our goal is to provide a proof-of-concept for blockchain-coordinated robot swarms, we did not strive for the best performance by fine-tuning algorithm parameters. For example, the approach could be extended and improved with more sophisticated outlier detection methods. Since smart contracts are Turing-complete, any outlier detection method is in principle implementable; in practice, however, one should choose a lightweight algorithm with a low run-time. Another aspect to consider is the operability of the approach in a dynamic environment. In the current implementation, the smart contract obtains a rough estimate in the first escrow round and then narrows down the collective estimate; a sudden change in the environment (e.g., the color of all the tiles is suddenly inverted) could lead to a dead-end situation, where all sensor readings in future escrow rounds are discarded by the outlier detection mechanism. However, in an improved version of the smart contract, one could, for example, always accept a minimum number of sensor readings per escrow round—even if they are outliers—to prepare the algorithm for dynamic environments. It is important to note that there is no need for adapting the robots' controllers when changing the outlier detection method in the smart contract.

Although we selected a specific scenario and task (consensus reaching in collective estimation), this result is promising for the field of swarm robotics in general: using smart contracts as meta-controllers might facilitate the implementation of various other existing and novel swarm robotics applications. To list concrete examples, besides the presented collective decision-making scenario, we believe a blockchain-based approach might be useful in:

- *task-allocation scenarios*: e.g., in an area exploration scenario, a smart contract could identify unexplored areas and send control commands to different robots to explore these areas;
- *reputation management*: the tamper-proof nature of the blockchain allows for maintaining reputation values for the different robots;
- *lightweight machine learning algorithms*: e.g., a smart contract could serve as a database for sensor data and train a classification algorithm;
- *collective mapping*: parts of a map could be stored and aggregated in a smart contract;
- *robot-to-robot economies*: e.g., auction-based approaches, where the auction is executed via a smart contract;
- *robot-to-human economies*: e.g, people could pay robots for executing a task (monetization of jobs, leading to *robot as a service*) or, vice versa, people could offer rewards for the completion of a task.

In addition to considering other use cases, it is also possible to consider swarms composed of entities that are not robots. In this sense, this work can be seen as a stepping-stone for swarms composed of people, Internet-of-Things devices, and/or vehicles.

A blockchain is tamper-proof due to its decentralized consensus protocol that is able to maintain scarce resources in decentralized systems. In our research, we showed that these scarce “cryptotokens,” i.e., immutable units of exchange stored in the blockchain, can be used to prevent Sybil attacks in open robot swarms. A swarm is *open* when entities are free to join (e.g., because it turns out that the mission is too complex to be solved by a smaller swarm) and leave the swarm at any moment in time (e.g., because of a hardware failure). Sending a message via a blockchain is only possible when the sender spends some amount of cryptotokens. Hence, the number of messages a robot can send is limited and Sybil attacks can be prevented. This is of the utmost importance for many swarm robotics applications where a Sybil attack would undermine the swarm performance. For example, in voting scenarios without Sybil attack protection, an attacker would be able to achieve the majority; and in sensor fusion scenarios, an attacker would be able to gravely bias the swarm estimate. These attacks do not require sophisticated programming skills and are hard to prevent in decentralized systems (Borisov, 2006). The most used means of preventing such attacks are centralized cryptographic authentication or password authentication. In our case, this would have meant that at the beginning of a simulation run, each robot would have received a list of public keys that are seen as trusted entities and would only have accepted a message from another robot if the message was signed by one of the trusted robots. However, this would entail the common disadvantages of centralized systems, such as the presence of a single point of failure at the moment when the list of public keys is created and distributed and reduced flexibility, since every robot must be identified before deployment and adding robots at run time would not be possible. Therefore, basing the approach on centralized cryptographic authentication would restrict the applicability to *closed* robot swarms.

Finally, a blockchain serves as a tamper-proof audit log and keeps track of all relevant information from all robots over time. In real-world applications it may happen that only a single robot can be retrieved (e.g., only one robot might be physically reachable, or retrieving robots might be very expensive)^12^. However, the information written in its blockchain may be sufficient to reconstruct the complete course of the experiment. This information can be post-processed, e.g., outliers could even be detected after the end of the experiment. In addition, any other irregularities can be spotted and analyzed, e.g., for digital forensics.

### 7.2. Limitations

Our results clearly showed that the blockchain-based consensus protocol outperforms existing consensus protocols when Byzantine robots are present and that it is even needed when wishing to reach consensus in a decentralized manner under Sybil attack. While we can conclude that robot swarms are better off with blockchain technology, certain constraints need to be considered at the design stage before choosing to work with blockchain-controlled robot swarms.

A first possible issue is the fact that blockchains can introduce delays. Transactions first have to be mined to be considered by the smart contracts. Therefore, if fast reactions to messages are required, blockchains are not advisable. Instead, blockchains should be used for security-relevant data and should be combined with traditional local processing to yield hybrid approaches. Therefore, it is important to determine which information is security-relevant and should be processed via smart contracts (“on-chain”) and which information can be processed locally by single robots (“off-chain”).

Another possible issue is connected with blockchain technology's storage requirements. This was however not the case in our experiments, where the size of an escrow blockchain transaction was 148 Bytes and the total size of the blockchain (including auxiliary files) reached on average 6.8 MB after 1,000 s. During these 1,000 s, on average, 350 transactions were stored in the blockchain. To further test scalability, we conducted experiments with a run-time of 24 h with 20 robots. After the 24 h, the total size of the blockchain reached on average 33 MB. The blockchain size grows linearly after an initialization phase of approximately 6 h during which approximately one block is created per second; in the beginning, the network needs to adapt to the hash power in the network; after 6 h, one block is created approximately every 15 s. This time interval is the default in Ethereum, and could be changed if necessary. If we hypothesize robots with 16 GB of storage capacity—this is within the capacity of state-of-the-art swarm robotic platforms, such as the Pi-puck robot (Millard et al., 2017)—the storage would last for approximately 485 days.

Another aspect of scalability is the influence of the robot swarm size on the blockchain size. Adding more robots to the swarm might increase the blockchain size because a larger swarm might create more transactions. In the following calculation, we assume that 1,000 robots create 50 times more transactions than 20 robots and that each robot creates a transaction every 45 s. With these 1,000 robots, the upper limit for the estimated blockchain size would be 1.5 GB after 24 h. Please note that this calculation is just a rough approximation and that the study of scalability has other aspects that should be taken into account in future research, such as: (i) with a larger swarm size, it might suffice to create a transaction after longer intervals, reducing in this way the overall dimension of the blockchain; (ii) transactions could be aggregated or preprocessed before sending them to the blockchain; and (iii) since the PoW algorithm adapts to the hash power in the network, the number of mined blocks is largely independent of the number of robots, therefore, the space requirements for a greater number of robots will grow sublinearly.

In this article, we used a PoW-based consensus protocol. In contrast to popular opinion, PoW does not require sophisticated hardware and does not become necessarily harder over time. The difficulty of the mining puzzle depends on the total hash power in the network. Less powerful hardware leads to lower hash power. From a theoretical point of view, it would be possible to mine on a Kilobot (Rubenstein et al., 2014), which has an 8 MHz processor. In addition, it has been demonstrated that a variety of single board processors with arm processors (e.g., the Raspberry Pi) are able to mine and run Ethereum nodes^13^. If, however, an intruder can outperform the hash power of the remaining robots (51% attack), it can change the order of the transactions and decide whether or not transactions should be included in the blockchain. Therefore, the higher the hash power of the network, the more difficult it is to perform a 51% attack. In this article, we used 2.0 GHz and 1.8 GB of RAM so that Ethereum works “out-of-the-box,” as explained in section 4. In order to let Ethereum run on robots with more limited hardware such as those that we have recently acquired in our lab, we have created a modified version of Ethereum's source code^14^. With these modifications, Ethereum, including PoW, runs on the Pi-puck robots in our lab. By changing the initial difficulty specified in the genesis block, it is possible to establish a direct mapping for the time it takes to perform the PoW calculations from our simulations to the physical hardware.

A powerful intruder cannot forge signatures or change the logic implemented in a smart contract. Therefore, it depends on the context whether a PoW-based consensus protocol is adequate. If no powerful intruder is expected to enter the swarm (e.g., in an underwater exploration), PoW can be suitable: as long as the majority of robots acts according to the protocol, the data in the blockchain can be trusted. In robot swarm deployments, one might be concerned that the computational overhead required by PoW might lead to battery drain. However, preliminary results (not discussed in this article) show that the power consumption due to the blockchain mining activity is low and compatible with experimentation with a swarm of Pi-puck robots.

If the swarm is operating in an environment where reliable global communication is possible, PoW does not need to be run on the hardware of the robots. In this case, a custom blockchain network maintained by the robots is not necessary. Instead, a smart contract could be used in the main Ethereum network. Since the main Ethereum network is maintained by a decentralized network of computers, it does not pose a single point of failure. However, such a scenario would change some other aspects (e.g., the entry conditions for new robots) and would possibly have a stronger focus on economics (e.g., attacks would become expensive in terms of the “main Ethereum network” cryptocurrency, that has a certain market price), so we will leave it for future work. The scope of this article is strictly limited to swarm robotics to avoid any confusion with centralized multi-robot systems.

### 7.3. Future Work

In this article, we studied attacks at the collective estimation level by sending deceitful data. However, there is a difference between attacks at the collective estimation level and attacks at the blockchain level. Some attacks that can pose problems to decentralized systems, such as replay attacks, are naturally prevented by blockchain technology. Yet, there are potential blockchain-level attacks in robot swarms: for example, clustering of malicious robots to perform a majority attack. In order to prevent majority attacks, a flocking algorithm may guarantee a certain degree of connectivity and help to avoid local robot clusters that have different blockchain forks. As an additional procedure to manage blockchain forks, the number of confirmations (i.e., the number of blocks after the block number that contains a certain transaction) can serve as a metric indicating how probable it is that a transaction stays in a specific block.

The robustness of the blockchain approach to much sparser connectivity is an open research topic that we plan to address in future research. As described in section 2.1, transactions stay valid and can be included in the blockchain after days of disconnectivity or after a blockchain fork gets discarded (they then become unconfirmed transactions again that can be included in later blocks). However, the longer the robot clusters stay disconnected, the higher the risk that they base a decision on a blockchain fork that is not the longest blockchain. There are several strategies to address this issue. One option is to increase the average time between mined blocks (block time) via a different difficulty setting. This will introduce delays but reduce the risk that decisions are based on non-final information. Further possibilities are aggregation algorithms to guarantee a certain connectivity; or “messenger robots” that can move faster (e.g., UAVs) and bring together different blockchain information. The robot that we are currently planning to use (the Pi-puck) has a Wi-Fi speed of up to 72 Mbps. Therefore, if a robot in the studied scenario would join the swarm after 20 min, it could download the blockchain within a few seconds from other robots. In future research, we will measure the relationship between the time two components of the swarm were disconnected and the time it takes to re-synchronize the blockchain across the disconnected robots afterwards.

In future work, we plan to transfer the system to heterogeneous robot swarms where some of the robots might have very different computational capabilities. In such a heterogeneous robot swarm, the overhead of blockchain technology could be delegated to the more powerful robots. For example, a swarm of smaller Kilobots could report back to larger Pi-puck robots at certain intervals. The Pi-puck robots could store the blockchain and perform the PoW, while the Kilobots just create transactions.

Another option to bring blockchain technology to robots of any size is to use a different blockchain framework. In the last couple of years, blockchain technology has experienced dramatic development. While at the start of this research work Ethereum was the only fully-developed blockchain-based smart contract platform, there are now more than a dozen smart contract platforms. These frameworks differ, among other aspects, in terms of their computational requirements, consensus protocol, scalability, robustness, speed, and use cases. The nature of, for example, public-key cryptography, transactions, and smart contracts, is largely independent of the used consensus protocol. Therefore, our work can serve as a basis for studying other blockchain frameworks, such as, Hyperledger Sawtooth^15^, Cardano^16^, and Tezos^17^ in the context of robot swarms. By means of these blockchain frameworks, we intend to compare alternatives to the *Proof-of-Work*-based consensus protocol on both the physical robots and via the ARGoS-Blockchain interface in future work. We plan to study *Proof-of-Stake* (already implemented in some existing blockchain protocols), *Proof-of-Sensing* (only robots that can produce a certain sensory output can send or validate transactions), or even *Proof-of-physical-Work* (only robots that can prove that they have performed physical work, such as collecting an item can send or validate transactions).

## 8. Conclusions

In this article, our goal was to compare consensus protocols used in swarm robotics with regard to their resilience to Byzantine robots. We showed that existing consensus protocols can easily fail in the presence of Byzantine robots. With the developed ARGoS-blockchain interface, we provide a framework for secure robot swarm coordination via blockchain-based smart contracts as “meta-controllers.” Blockchain technology makes sure that every robot runs the same code, that the code is executed exactly as specified, that the robots come to a consensus regarding the outcome of the execution, and that there is not a single point of failure. Blockchains prevent Sybil attacks via their scarce cryptocurrency that limits the number of transactions a robot can send. Additionally, the blockchain is able to securely store critical events. This decentralized log can then be used to evaluate the quality of experiments and to spot irregularities.

Blockchain-controlled robot swarms must meet certain computational and memory requirements. Compared to Internet-based blockchain networks, in robot swarms, the computational capacities are limited, the delays can be much longer, and failing entities are more probable due to rough environmental conditions or flat batteries. While we discussed these characteristics, we do not question the fact that there are still many open challenges for blockchain-based swarm robotics. Nevertheless, we are convinced that the synthesis of these two technologies offers unprecedented possibilities and that the various challenges can gradually be addressed. In this article we have shown that blockchain-based smart contracts are a promising and versatile tool to address security issues in swarm robotics. If we ever want robot swarms to be deployed in the real world, we need to start preparing them to the possible presence of Byzantine robots: the work we have presented is a first step in this direction.

## Data Availability Statement

The datasets generated for this study can be found in the IRIDIA—Supplementary Information (ISSN: 2684-2041) at http://iridia.ulb.ac.be/supp/IridiaSupp2019-009/.

## Author Contributions

All authors contributed to the conceptualization of this research and the setup of the experiments. VS implemented the software and conducted and analyzed the experiments. In addition, he wrote the first draft of this manuscript. EC and MD gave critical feedback, revised the article, and contributed to the final manuscript.

## Conflict of Interest

The authors declare that the research was conducted in the absence of any commercial or financial relationships that could be construed as a potential conflict of interest. The handling editor declared a past co-authorship with one of the authors MD.

## Footnotes

^1^Note that in this article, as said before, all experiments are run in simulation. Porting our system on real robots will be the subject of future work.

^2^https://github.com/Pold87/ARGoS-Blockchain-interface

^3^https://github.com/Capgemini-AIE/ethereum-docker, accessed on November 6, 2019

^4^The interface uses shell scripts, since, during development, it became evident that they are executed much faster than other Ethereum APIs.

^5^This is a reasonable choice as a robot's computer could easily have such characteristics. It is also a convenient choice because on a computer with 2.0 GHz and 1.8 GB of RAM, Ethereum works “out-of-the-box,” without any modifications; therefore, any interested user can obtain the most recent release of Ethereum from the official depository and use it with our publicly available ARGoS-Blockchain interface.

^6^Since we do not use the main Ethereum network but a custom network maintained by the robots, these ethers have value only within the robot swarm.

^7^In a real-world scenario, determining a suitable price might be difficult. However, there is a simple but effective remedy: instead of sending a transaction every 45 s, robots could send a transaction as soon as they have enough ether to create a transaction. We did not implement this “remedy” because we wanted the three compared approaches to differ in as few aspects as possible.

^8^https://github.com/Pold87/robot-swarms-need-blockchain-classical

^9^https://github.com/Pold87/robot-swarms-need-blockchain

^10^loess smoothing (Jacoby, 2000) is a non-parametric regression method to fit non-linear data. To do so, the loess algorithm performs local linear regressions via a weighted sliding-window approach. In other words, for each point in the dataset (the current focal point), it takes a subset of the whole dataset (in our case the 75% nearest neighbors) to calculate the least-squares fit. The higher the distance to the focal point, the lower the weight in the least-squares fit. We used the default settings as provided by the R programming language, described at https://www.rdocumentation.org/packages/stats/versions/3.6.2/topics/loess (accessed February 6, 2020).

^11^We set the maximum number of Byzantine robots to 7 since related literature usually considers a maximum of 33% Byzantine agents.

^12^If one has, however, the possibility to choose between different robots, one may select the robot with the longest blockchain to make sure that the chain is selected where the highest number of participants contributed to the Proof-of-Work.

^13^http://ethembedded.com/, accessed on September 17, 2019.

^14^The necessary modifications can be found at http://iridia.ulb.ac.be/supp/IridiaSupp2019-009/Ethereum-on-Pi-puck/. Ethereum's default mining algorithm—an extension of the basic PoW algorithm, as for example used in Bitcoin—includes a memory-hard problem to make it resistant to specialized mining hardware using ASICs. This is done via the generation of a data structure called DAG which requires more than 1.0 GB of RAM (for a description of the DAG, see https://github.com/ethereum/wiki/wiki/ethash-dag, accessed on February 16, 2020). With the described modifications, the RAM requirements can be reduced to a few MB.

^15^https://sawtooth.hyperledger.org/ (accessed September 12, 2019).

^16^https://www.cardano.org/en/home/ (accessed on September 12, 2019).

^17^https://tezos.com/ (accessed September 12, 2019).

## References

[B1] Beal J. (2016). Trading accuracy for speed in approximate consensus. Knowl. Eng. Rev. 31, 325–342. 10.1017/S0269888916000175

[B2] Borisov N. (2006). “Computational puzzles as Sybil defenses,” in Proceedings of the Sixth IEEE International Conference on Peer-to-Peer Computing (P2P '06) (Los Alamitos, CA: IEEE Press), 171–176.

[B3] Brown A. Franken P. Bonner S. Dolezal N. Moross J. (2016). Safecast: successful citizen-science for radiation measurement and communication after Fukushima. J. Radiol. Protect. 36, S82–S101. 10.1088/0952-4746/36/2/S82 27270965

[B4] Buterin V. (2014). A Next-Generation Smart Contract and Decentralized Application Platform. Ethereum Project White Paper. Technical Report. Available online at: https://github.com/ethereum/wiki/wiki/White-Paper (Accessed July 18, 2019).

[B5] Castelló Ferrer E. (2016). The blockchain: a new framework for robotic swarm systems. arXiv:1608.00695v3. 10.1007/978-3-030-02683-7_77

[B6] Christensen A. L. O'Grady R. Birattari M. Dorigo M. (2008). Fault detection in autonomous robots based on fault injection and learning. Auton. Robots 24, 49–67. 10.1007/s10514-007-9060-9

[B7] Christensen A. L. O'Grady R. Dorigo M. (2009). From fireflies to fault-tolerant swarms of robots. IEEE Trans. Evol. Comput. 13, 754–766. 10.1109/TEVC.2009.2017516

[B8] Crosby M. Pattanayak P. Verma S. Kalyanaraman V. (2016). Blockchain technology: beyond bitcoin. Appl. Innov. 2:71 10.1109/iCCECOME.2018.8658518

[B9] Dorigo M. Trianni V. Şahin E. Groß R. Labella T. H. Baldassarre G. (2004). Evolving self-organizing behaviors for a swarm-bot. Auton. Robots 17, 223–245. 10.1023/B:AURO.0000033973.24945.f3

[B10] Douceur J. R. (2002). “The Sybil attack,” in 1st International Workshop on Peer-to-Peer systems, Vol. 2429 of Lecture Notes in Computer Science, eds P. Druschel, F. Kaashoek, and A. Rowstron (Berlin; Heidelberg: Springer), 251–260.

[B11] Elhage N. Beal J. (2010). “Laplacian-based consensus on spatial computers,” in Proceedings of the 9th International Conference on Autonomous Agents and MultiAgent Systems (AAMAS 2010) (Richland, SC: International Foundation for Autonomous Agents and Multiagent Systems), 907–914.

[B12] Fernandes M. Alexandre L. A. (2019). Robotchain: using Tezos technology for robot event management. Ledger 4(Suppl. 1). 10.5195/ledger.2019.175

[B13] Ferrante E. Turgut A. E. Huepe C. Stranieri A. Pinciroli C. Dorigo M. (2012). Self-organized flocking with a mobile robot swarm: a novel motion control method. Adapt. Behav. 20, 460–477. 10.1177/1059712312462248

[B14] Gil S. Kumar S. Mazumder M. Katabi D. Rus D. (2017). Guaranteeing spoof-resilient multi-robot networks. Auton. Robots 41, 1383–1400. 10.1007/s10514-017-9621-5

[B15] Guerrero-Bonilla L. Prorok A. Kumar V. (2017). Formations for resilient robot teams. IEEE Robot. Autom. Lett. 2, 841–848. 10.1109/LRA.2017.2654550

[B16] Gupta V. Langbort C. Murray R. M. (2006). “On the robustness of distributed algorithms,” in Proceedings of the 45th IEEE Conference on Decision and Control (Piscataway, NJ: IEEE Press), 3473–3478.

[B17] Higgins F. Tomlinson A. Martin K. M. (2009). “Survey on security challenges for swarm robotics,” in Proceedings of the Fifth International Conference on Autonomic and Autonomous Systems (Piscataway, NJ: IEEE Press), 307–312.

[B18] Jacoby W. G. (2000). Loess: a nonparametric, graphical tool for depicting relationships between variables. Elect. Stud. 19, 577–613. 10.1016/S0261-3794(99)00028-1

[B19] Lamport L. Shostak R. Pease M. (1982). The Byzantine generals problem. ACM Trans. Programm. Lang. Syst. 4, 382–401.

[B20] LeBlanc H. J. Zhang H. Koutsoukos X. Sundaram S. (2013). Resilient asymptotic consensus in robust networks. IEEE J. Select. Areas Commun. 31, 766–781. 10.1109/JSAC.2013.130413

[B21] Lopes V. Alexandre L. A. (2019). “Detecting robotic anomalies using robotchain,” in IEEE International Conference on Autonomous Robot Systems and Competitions (ICARSC 2019) (Piscataway, NJ: IEEE Press), 1–6.

[B22] McAbee A. Tummala M. McEachen J. (2019). “Military intelligence applications for blockchain technology,” in Proceedings of the 52nd Hawaii International Conference on System Sciences (Honolulu, HI: ScholarSpace), 6031–6040.

[B23] Merkel D. (2014). Docker: lightweight linux containers for consistent development and deployment. *Linux J*. 2014. Available online at: https://www.linuxjournal.com/content/docker-lightweight-linux-containers-consistent-development-and-deployment

[B24] Millard A. G. Joyce R. Hilder J. A. Fleşeriu C. Newbrook L. Li W. (2017). “The pi-puck extension board: a Raspberry Pi interface for the e-puck robot platform,” in 2017 IEEE/RSJ International Conference on Intelligent Robots and Systems (IROS) (Los Alamitos, CA: IEEE Press), 741–748.

[B25] Millard A. G. Timmis J. Winfield A. F. T. (2014). “Towards exogenous fault detection in swarm robotic systems,” in Towards Autonomous Robotic Systems - Proceedings of TAROS 2013 - 14th Annual Conference, Vol. 8069 of Lecture Notes in Computer Science (Cham: Springer), 429–430.

[B26] Montes de Oca M. A. Ferrante E. Scheidler A. Pinciroli C. Birattari M. Dorigo M. (2011). Majority-rule opinion dynamics with differential latency: a mechanism for self-organized collective decision-making. Swarm Intell. 5, 305–327. 10.1007/s11721-011-0062-z

[B27] Nakamoto S. (2008). Bitcoin: A Peer-to-Peer Electronic Cash System. Technical Report. Available online at: https://bitcoin.org/bitcoin.pdf (Accessed August 11, 2018).

[B28] Nishida Y. Kaneko K. Sharma S. Sakurai K. (2018). “Suppressing chain size of blockchain-based information sharing for swarm robotic systems,” in Proceedings of the Sixth International Symposium on Computing and Networking Workshops (CANDARW 2018) (Los Alamitos, CA: IEEE Press), 524–528.

[B29] Olfati-Saber R. Murray R. M. (2004). Consensus problems in networks of agents with switching topology and time-delays. IEEE Trans. Autom. Control 49, 1520–1533.

[B30] Pinciroli C. Trianni V. O'Grady R. Pini G. Brutschy A. Brambilla M. (2012). ARGoS: A modular, parallel, multi-engine simulator for multi-robot systems. Swarm Intell. 6, 271–295. 10.1007/s11721-012-0072-5

[B31] Primiero G. Tuci E. Tagliabue J. Ferrante E. (2018). “Swarm attack: a self-organized model to recover from malicious communication manipulation in a swarm of simple simulated agents,” in Swarm Intelligence – Proceedings of ANTS 2018 – Eleventh International Conference, eds M. Dorigo, M. Birattari, C. Blum, A. L. Christensen, A. Reina, and V. Trianni (Cham: Springer), 213–224.

[B32] Reina A. Dorigo M. Trianni V. (2014). “Collective decision making in distributed systems inspired by honeybees behaviour,” in Proceedings of the 13th International Conference on Autonomous Agents and MultiAgent Systems (AAMAS 2014), eds A. Lomuscio, P. Scerri, A. Bazzan, and M. Huhns (Richland, SC: International Foundation for Autonomous Agents and Multiagent Systems), 1421–1422.

[B33] Reina A. Valentini G. Fernández-Oto C. Dorigo M. Trianni V. (2015). A design pattern for decentralised decision making. PLoS ONE 10:e0140950. 10.1371/journal.pone.0140950 26496359 PMC4619747

[B34] Roumeliotis S. I. Sukhatme G. S. Bekey G. A. (1998). “Sensor fault detection and identification in a mobile robot,” in Proceedings of the 1998 IEEE/RSJ International Conference on Intelligent Robots and Systems. Innovations in Theory, Practice and Applications (Cat. No. 98CH36190), Vol. 3 (New York, NY: IEEE Press), 1383–1388.

[B35] Rubenstein M. Ahler C. Hoff N. Cabrera A. Nagpal R. (2014). Kilobot: a low cost robot with scalable operations designed for collective behaviors. Robot. Auton. Syst. 62, 966–975. 10.1016/j.robot.2013.08.006

[B36] Saldaña D. Prorok A. Sundaram S. Campos M. F. M. Kumar V. (2017). “Resilient consensus for time-varying networks of dynamic agents,” in Proceedings of the American Control Conference (ACC) (Piscataway, NJ: IEEE Press), 252–258.

[B37] Sargeant I. Tomlinson A. (2016). “Maliciously manipulating a robotic swarm,” in Proceedings of ESCS'16 – The 14th International Conference on Embedded Systems, Cyber-Physical Systems, & Applications (Bogart, GA: CSREA Press), 122–128.

[B38] Saulnier K. Saldaña D. Prorok A. Pappas G. J. Kumar V. (2017). Resilient flocking for mobile robot teams. IEEE Robot. Autom. Lett. 2, 1039–1046. 10.1109/LRA.2017.2655142

[B39] Schmickl T. Thenius R. Moeslinger C. Radspieler G. Kernbach S. Szymanski M. (2009). Get in touch: cooperative decision making based on robot-to-robot collisions. Auton. Agents Multi-Agent Syst. 18, 133–155. 10.1007/s10458-008-9058-5

[B40] Soysal O. Sahin E. (2005). “Probabilistic aggregation strategies in swarm robotic systems,” in Proceedings of the 2005 IEEE Swarm Intelligence Symposium (SIS 2005) (Piscataway, NJ: IEEE Press), 325–332.

[B41] Strobel V. Castelló Ferrer E. Dorigo M. (2018). “Managing Byzantine robots via blockchain technology in a swarm robotics collective decision making scenario,” in Proceedings of the 17th International Conference on Autonomous Agents and MultiAgent Systems (AAMAS 2018), eds M. Dastani, G. Sukthankar, E. André, and S. Koenig (Richland, SC: International Foundation for Autonomous Agents and Multiagent Systems), 541–549.

[B42] Strobel V. Dorigo M. (2018). “Blockchain technology for robot swarms: a shared knowledge and reputation management system for collective estimation,” in Swarm Intelligence – Proceedings of ANTS 2018 – Eleventh International Conference, Vol. 11172 of Lecture Notes in Computer Science, eds M. Dorigo, M. Birattari, C. Blum, A. L. Christensen, A. Reina, and V. Trianni (Cham: Springer), 425–426.

[B43] Szabo N. (1997). Formalizing and securing relationships on public networks. First Monday 2 10.5210/fm.v2i9.548

[B44] Tarapore D. Christensen A. L. Timmis J. (2017). Generic, scalable and decentralized fault detection for robot swarms. PLoS ONE 12:e182058. 10.1371/journal.pone.0182058 28806756 PMC5555700

[B45] Tarapore D. Lima P. U. Carneiro J. Christensen A. L. (2015). To err is robotic, to tolerate immunological: fault detection in multirobot systems. Bioinspir. Biomim. 10:016014. 10.1088/1748-3190/10/1/016014 25642825

[B46] Tarapore D. Timmis J. Christensen A. (2019). Fault detection in a swarm of physical robots based on behavioral outlier detection. IEEE Trans. Robot. 35:1–7. 10.1109/TRO.2019.2929015

[B47] Valentini G. Brambilla D. Hamann H. Dorigo M. (2016a). “Collective perception of environmental features in a robot swarm,” in Swarm Intelligence – Proceedings of ANTS 2016 – Tenth International Conference, Vol. 9882 of Lecture Notes in Computer Science (Cham: Springer), 65–76.

[B48] Valentini G. Ferrante E. Dorigo M. (2017). The best-of-n problem in robot swarms: formalization, state of the art, and novel perspectives. Front. Robot. AI 4:9 10.3389/frobt.2017.00009

[B49] Valentini G. Ferrante E. Hamann H. Dorigo M. (2016b). Collective decision with 100 Kilobots: speed versus accuracy in binary discrimination problems. Auton. Agents Multi-Agent Syst. 30, 553–580. 10.1007/s10458-015-9323-3

[B50] Winfield A. F. T. Nembrini J. (2006). Safety in numbers: fault tolerance in robot swarms. Int. J. Modell. Identif. Control 1, 30–37. 10.1504/IJMIC.2006.008645

[B51] Xiao L. Boyd S. Lall S. (2005). “A scheme for robust distributed sensor fusion based on average consensus,” in The Fourth International Symposium on Information Processing in Sensor Networks (IPSN 2005) (Piscataway, NJ: IEEE Press), 63–70.

[B52] Zikratov I. Maslennikov O. Lebedev I. Ometov A. Andreev S. (2016). “Dynamic trust management framework for robotic multi-agent systems,” in Proceedings of the 12th International Conference on Next Generation Teletraffic and Wired/Wireless Advanced Networking (NEW2AN 2016), and the 5th Conference on Internet of Things and Smart Spaces (ruSMART 2016), eds O. Galinina, S. Balandin, and Y. Koucheryavy (Cham: Springer), 339–348.

